# Enhanced impact resistance of novel sustainable preplaced aggregate geopolymer concrete reinforced with steel mesh and 5D fibers

**DOI:** 10.1038/s41598-025-14281-9

**Published:** 2025-08-12

**Authors:** Mostafa Samadi, G. Murali, Leong Sing Wong, Marzena Kurpińska, Hakim S. Abdelgader, Isyaka Abdulkadir, Nor Hasanah Abdul Shukor Lim, Siva Avudaiappan, Mohamed Abdellatief

**Affiliations:** 1 Institute of Energy Infrastructure, Universiti Tenaga Nasional, Jalan IKRAM-UNITEN, 43000 Kajang, Selangor Malaysia; 2 Centre for Promotion of Research, Graphic Era (Deemed to be University), Clement Town, Dehradun, India; 3 Faculty of Civil and Environmental Engineering, Gdansk University of Technology, Gdansk, Poland; 4 Civil Engineering Department, Faculty of Engineering, University of Tripoli, Tripoli, Libya; 5 Faculty of Civil Engineering, UTM Construction Research Center (CRC), University Teknologi Malaysia, Blok C09, 81310 Johor Darul Takzim, Malaysia; 6 Departamento de Ciencias de la Construcción, Facultad de ciencias de la construccion y ordenamiento territorial, Universidad Tecnológica Metropolitana, Santiago, Chile; 7 Department of Civil Engineering, Higher Future Institute of Engineering and Technology in Mansoura, Mansoura, Egypt

**Keywords:** Impact strength, 5D fibers, Steel wire mesh, Geopolymer grout, Preplaced aggregate concrete, Microstructure, Civil engineering, Composites

## Abstract

The rising demand for sustainable concrete stems from resource scarcity, environmental concerns, and structural performance needs. Preplaced Aggregate Concrete (PAC) improves durability and efficiency but requires alternative binders to lessen dependence on Portland cement. This study explores the formulation of a sustainable geopolymer grout, incorporating red clay, slag, and fly ash, to address these concerns while promoting the reutilization of industrial by-products. This study investigates the synergistic integration of steel wire mesh (SWM) and advanced 5D steel fibers (2.5% by volume) to improve the impact resistance of PAC. Five distinct mesh sizes (M40, M30, M20, M10 and M5), with diameters ranging from 75 mm to 150 mm at 25 mm intervals, were strategically placed at the mid-height of the PAC. A total of 42 mixing combinations were developed and categorized into 10 groups based on variations in steel wire mesh sizes and fiber configurations. All specimens underwent evaluation using the drop-weight impact test in conformity with ACI Committee guidelines. The innovation combines sustainable geopolymer binders with hybrid reinforcement, creating a concrete system with enhanced impact strength. Microstructural analysis was also performed on the geopolymer grout used in PAC. SWM integration in PAC notably enhances failure impact number, especially with larger diameters (150 mm), while first crack sees only slight improvement. Combining SWM with steel fibers consistently boosts both initial crack and failure by improving crack control and energy absorption. Larger SWM diameters (e.g., 150 mm) lead to more distributed failure patterns and better energy dissipation than smaller diameters (e.g., 75 mm).

## Introduction

The pursuit of sustainability in the construction sector is impeded by critical challenges such as the depletion of natural resources, escalating carbon emissions, and the demand for materials capable of withstanding intricate structural loads^1^. Ordinary Portland Cement (OPC), the predominant binder in concrete, significantly impacts environmental sustainability due to its high energy requirements and substantial CO₂ emissions during production^2,3^. Therefore, identifying alternative binders that address these environmental concerns while meeting the rigorous performance requirements of modern construction practices is an urgent priority^4^. In addition to environmental concerns, the structural performance of Preplaced Aggregate Concrete (PAC) under extreme conditions, such as impact loading, remains a critical area of investigation^5,6^. While conventional PAC systems demonstrate considerable strength^7^, there is a need for advancements in impact resistance to broaden their applicability in construction, particularly for blast-resistant structures and industrial flooring^8^. PAC is an innovative form of concrete that departs from conventional methods by arranging coarse aggregates in the mold before injecting a cementitious grout to fill the voids. This eliminates traditional mixing and placement, making PAC a highly efficient and durable construction material. Its unique production process minimizes segregation and bleeding, ensures uniform strength, reduces reliance on cementitious binders, and lowers environmental impact^9^. In addition, the preplacement technique enhances aggregate-grout bonding, resulting in superior compressive strength and impact resistance^10^. Lv et al.^11^ illustrated that utilizing PAC as a substitute for conventional concrete in construction practices could achieve a reduction of 15–20% in cement consumption. This substitution presents substantial environmental advantages while simultaneously delivering significant economic benefits.

Numerous research started to investigate the mechanical properties of PAC using cement grout^6^. Das et al.^12^ conducted a detailed analysis of PAC, focusing on its properties when cement was partially substituted by silica fume (SF) in proportions ranging from 0 to 10% and ground granulated blast furnace slag (GGBS) in percentages varying between 0% and 40%. When contrasted with PAC which does not contain supplementary cementitious materials, PAC including 40% GGBS and 10% SF exhibits compressive and splitting tensile strengths that are either comparable to or surpass those of the conventional mix. According to the observations of Ichino et al.^8^, when the compressive strength surpasses 17.6 MPa, the crater depth and diameter exhibit stabilization, displaying negligible variation despite subsequent increases in strength. At a compressive strength of 30.6 MPa, the spall diameter and depth attain their minimum values. However, with further increments in compressive strength, both spall depth and diameter show a marginal increase. These findings underscore the necessity of exercising equivalent levels of precision and care in the design and construction of protective structures utilizing high-strength PAC, akin to those employing high-strength conventional concrete. Tuyan et al.^13^ proposed an innovation of environmentally friendly PAC grout using alkali-activated slag. The findings revealed that an increase in the Na_2_O concentration and the silicate modulus markedly enhanced the compressive strength of the grout formulations. Alkali-activated slag grout mixtures, characterized by optimal fresh-state performance, demonstrated compressive strength values within the range of 37 to 56 MPa, confirming their potential suitability for structural engineering applications. Tuyan et al.^14^ documented that preplaced aggregate concrete mixtures, formulated with 4% Na_2_O and a silicate modulus of 1.2, exhibited an elastic modulus of 30.2 GPa, a tensile strength of 3.5 MPa and compressive strength of 34.5 MPa. These results affirm the potential for developing structurally robust alkali-activated PAC.

Impact resistance testing is crucial for evaluating geopolymer concrete’s ability to withstand sudden loads such as debris impact, seismic events, or drop-weight shocks, common in infrastructure and protective structures^15^. These tests reveal insights into energy absorption, crack propagation, and post-impact behavior, supporting the development of tougher, more ductile materials. Incorporating fibers like steel, polypropylene, or basalt has been shown to significantly enhance GPC’s impact resistance, addressing its brittle nature^16–18^. As GPC sees growing use in structural and prefabricated elements, understanding its impact performance is essential for ensuring safety and durability^19^. Numerous studies have investigated the impact resistance of concrete reinforced with fibers and steel wire mesh. For instance, Nehdi et al.^20^ found that PAC mixtures incorporating 1% and 2% short steel fibers showed compressive strength improvements of approximately 14% and 18%, respectively, relative to control specimens without steel fibers. The inclusion of 4% and 6% short steel fibers in PAC exhibited compressive strength enhancements of about 9% and 26%, respectively, compared to PAC incorporating 2% steel fibers. This improvement can be ascribed to the enhanced resistance to crack initiation and spread facilitated by the higher steel fiber content, which consequently led to an rise in compressive strength^21,22^.

According to Ma et al.^23^, the integration of steel wire mesh (SWM) into the test specimens resulted in a substantial improvement in structural performance. Specifically, SWM incorporation delayed the onset of cracking and increased the cracking strength by as much as 23.63%. Furthermore, the specimens exhibited a pronounced enhancement in energy dissipation capacity, with a peak improvement reaching 940.42%, indicating a marked increase in their ability to absorb and dissipate mechanical energy under loading conditions. Lu et al.^24^ reported that applying SWM changed the failure mode from brittle to ductile and increased the ultimate flexural capacity of jointed specimens by 196% compared to those without interface treatment. Li et al.^25^ reported that hybrid reinforcement with steel fibers and wire mesh enhances shear resistance and ductility. Samadi et al.^26^ found that integrating SWM into geopolymer based-PAC enhances fracture toughness by controlling crack propagation, improving tensile strength, and promoting ductile failure. The combined use of SWM and steel fibers increases shear resistance and crack-bridging capacity, resulting in improved load-bearing performance. Karthikeyan et al.^27^ highlighted that steel fibers exhibit significantly greater efficiency in improving the impact performance of PAC compared to glass fiber mesh (GFM). The analysis of impact energies demonstrated that PAC specimens reinforced with 3% steel fibers achieved substantial improvements relative to non-fibrous specimens, with cracking impact numbers increasing by 210–409% and failure impact numbers escalating by 1032–3108%. These advancements notably surpassed the enhancements attained with GFM insertion, reaffirming the superior efficiency of steel fibers in optimizing the impact resistance of PAC. Previous studies have documented the incorporation of various mesh types, including GFM^28^, GFM combined with multi-walled carbon nanotubes^29^, textile fiber mesh^30^, and multiple GFM placements^31^, within PAC, all of which demonstrated enhanced impact strength when fibers were included. Nevertheless, significant research gaps persist regarding the performance of mesh insertions when used with alternative grouting materials, particularly geopolymer grout, in enhancing impact strength.

### Red clay in geopolymer concrete

**Fig. 1:**
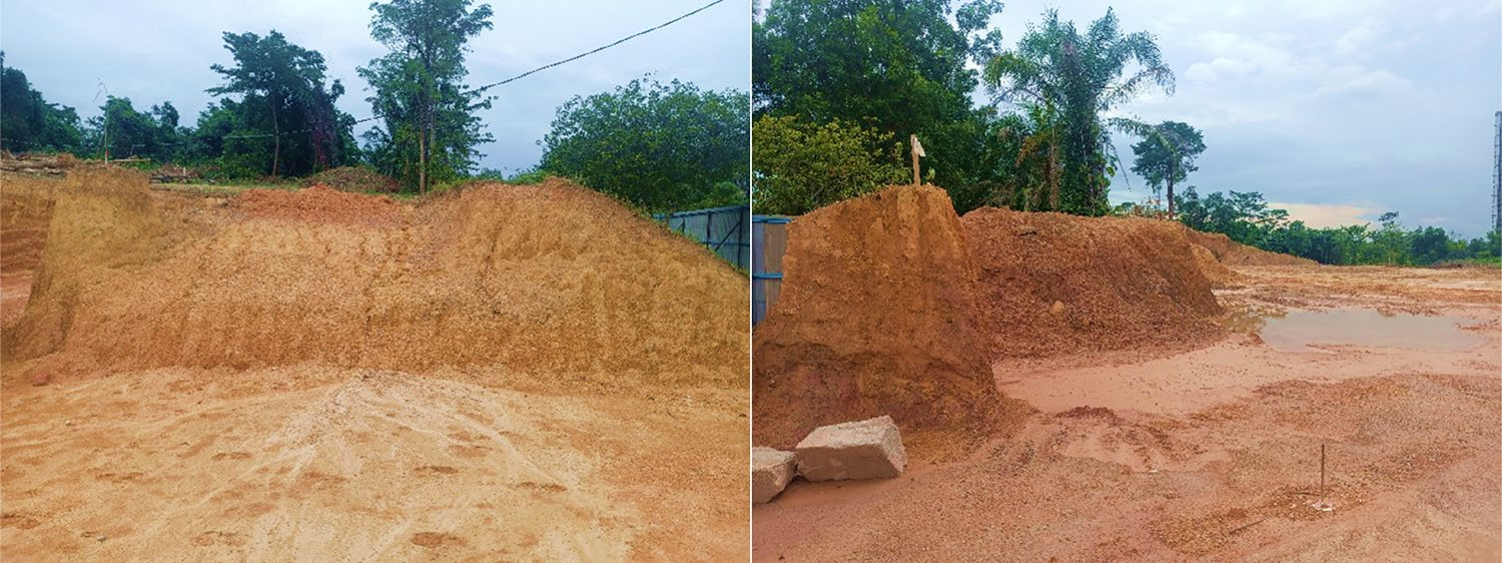
Natural source of red clay.

Geopolymer concrete represents a sustainable alternative to conventional Portland cement-based concrete, offering significant environmental advantages, and utilizes abundant materials such as red clay (RC), slag^32^, and fly ash^33^. The RC rich in silica and alumina, forms durable geopolymer binders when activated with alkaline solutions and available materials in nature (Fig. 1). Slag, a steel production by-product, enhances concrete’s mechanical properties and durability through its pozzolanic reaction. Fly ash, a coal combustion by-product, offers high pozzolanic reactivity, fine particles, and elevated silica content, making it ideal for use in geopolymer binders. Kumar and Kumar^34^ stated that fly ash-based geopolymer mixtures exhibited an initial setting time of 210 min and a final setting time of 310 min. The addition of RC up to 15% by weight reduced the setting time of the geopolymer. However, further increases in RC content resulted in a slower setting time. The RC content had a pronounced effect on both the strength and heavy metal ion adsorption capacity of red clay-GGBS-based geopolymer pervious concrete. As the RC proportion increased from 0 to 50% by weight, the compressive strength decreased, while the capacity for heavy metal ion adsorption improved significantly^35^. Guo et al.^36^ stated that the incorporation of red clay adversely affected the compressive strength and tensile properties of high-ductility geopolymer composites, with strength parameters inversely correlated to red clay dosage. The composite incorporating 30 wt% red clay exhibited a tensile ductility of 3.51%, a compressive strength of 48.7 MPa, and a tensile strength of 2.40 MPa, as determined through experimental evaluation.

Raza et al.^37^ identified the optimal geopolymer concrete composition for maximum compressive strength as comprising 15% calcium sulfate dihydrate, 45% GGBS and 40% calcined red clay. X-ray diffraction (XRD) analysis confirmed the existence of characteristic peaks corresponding to calcite, quartz, and portlandite within the mixtures. Notably, samples with elevated red clay content exhibited a more pronounced portlandite peak. Xu et al.^38^ demonstrated that the synergistic incorporation of nano-SiO_2_ and steel fibers significantly improves the microstructural density of the red clay-based geopolymer gel as well as the interfacial transition zone between the matrix and fibers. This enhancement directly contributes to improved mechanical strength. Xiaoshuang et al.^39^ demonstrated that fly ash–red clay-based mortar achieved flexural strength of 15.66 MPa and compressive strength of 69.06 MPa when the GGBS content was 50% and the NaOH concentration was 10 M. Increasing the GGBS content in geopolymer mortar enhances flexural and compressive strengths by introducing more CaO, which facilitates the development of denser C-S-H gel. Alessio Occhicone et al.^40^ investigated the development of geopolymer paste incorporating red clay and slag. Their findings revealed that, after 28 days of curing, the paste achieved a compressive strength of 70 MPa with a 50% red clay substitution and 50 MPa with a 70% substitution, demonstrating the impact of RC content on mechanical performance. Ghosh and Ransinchung^41^ reported that fly ash-RC geopolymer concrete cured under ambient conditions attained a maximum compressive strength of 31.5 MPa and a maximum flexural strength of 4.32 MPa, highlighting their mechanical performance under standard curing environments.

A multitude of research have reported the optimum use of RC in geopolymer concrete with fly ash, metakaolin and GGBS and their mechanical properties. However, none of the single studies was found in the literature regarding the impact resistance of RC-based geopolymer concrete with different fibers. In addition, geopolymer grout in PAC with steel wire mesh and 5D steel fiber is unexamined with these combinations. The integration of contemporary reinforcement methods, including steel wire mesh insertions at mid-height of specimens and 5D steel fibers, presents a promising strategy to significantly improve the mechanical characteristics of sustainable PAC, particularly its resistance to dynamic loads.

## Research significance

This investigation proposes an advanced hybrid reinforcement strategy that synergistically integrates geopolymer grout with steel wire mesh and 5D steel fibers. The study meticulously explores the combined influence of varying mesh dimensions (75–150 mm) and fiber volumes (2.5% by total concrete volume) on the impact resistance of PAC. The adoption of geopolymer grout fulfils a dual objective: diminishing dependence on OPC while fostering the reutilization of industrial by-products, thereby aligning with overarching global sustainability imperatives. The novelty of this work lies in its holistic approach to designing a high-performance, environmentally sustainable PAC system. By uniting eco-efficient binders, this study establishes a paradigm for minimizing the carbon footprint associated with concrete production. Comprehensive microstructural characterization of the geopolymer grout provides critical insights into its intrinsic properties and functional performance, further substantiating its applicability within PAC systems. This research addresses the urgent demand for materials that integrate ecological responsibility with exceptional structural capabilities, offering a transformative solution for advancing sustainability and functionality in the construction sector. It is significant because it improves the development of impact-resistant and eco-friendly PAC system while advancing innovative reinforcement method to promote high-performance concrete.

## Experimental methodology

### Materials

**Fig. 2:**
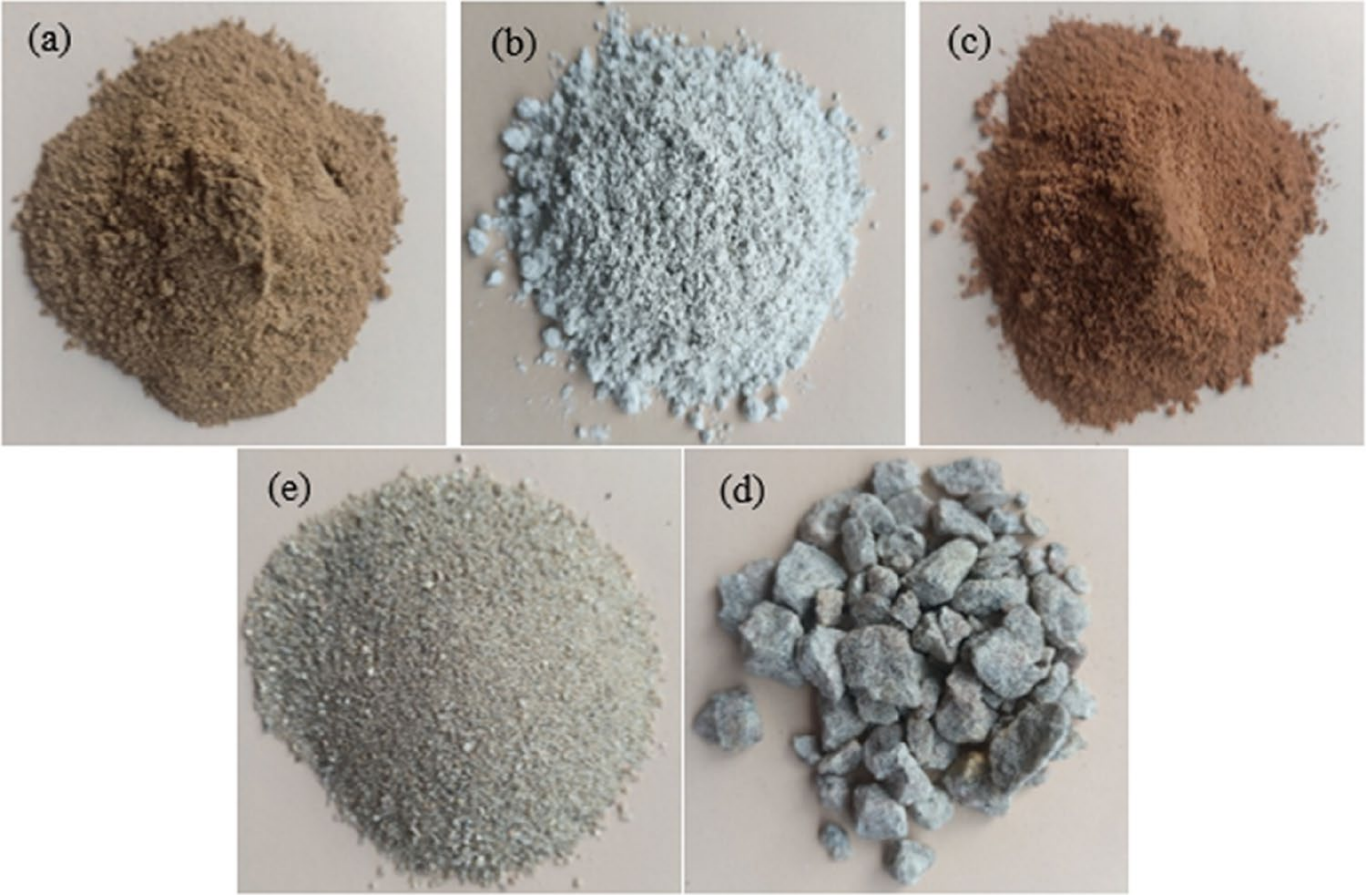
Raw materials (**a**) FA, (**b**) slag, (**c**) red clay, (**d**) fine aggregate and (**e**) coarse aggregate.

**Fig. 3:**
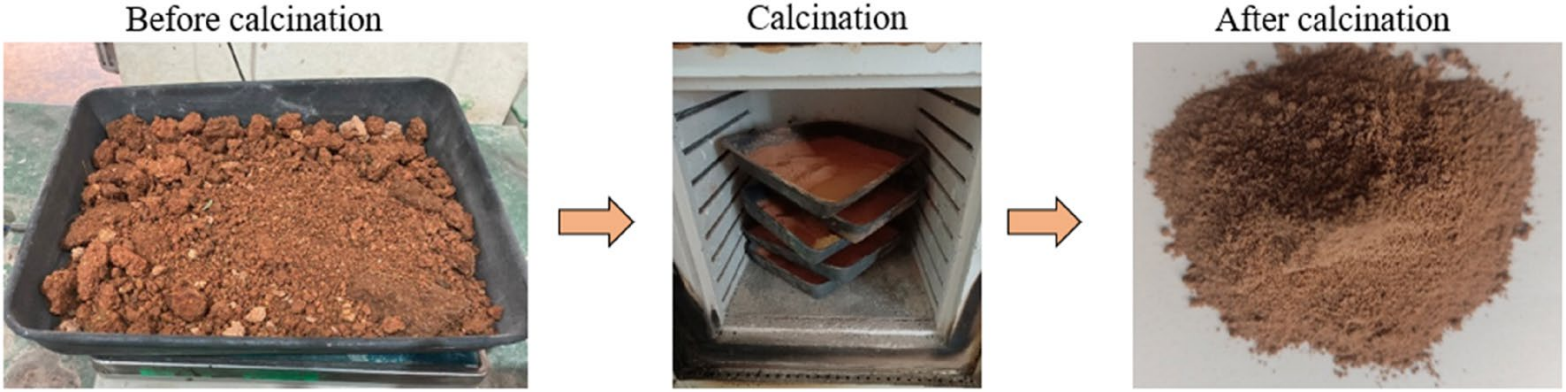
Calcination of red clay.

**Fig. 4:**
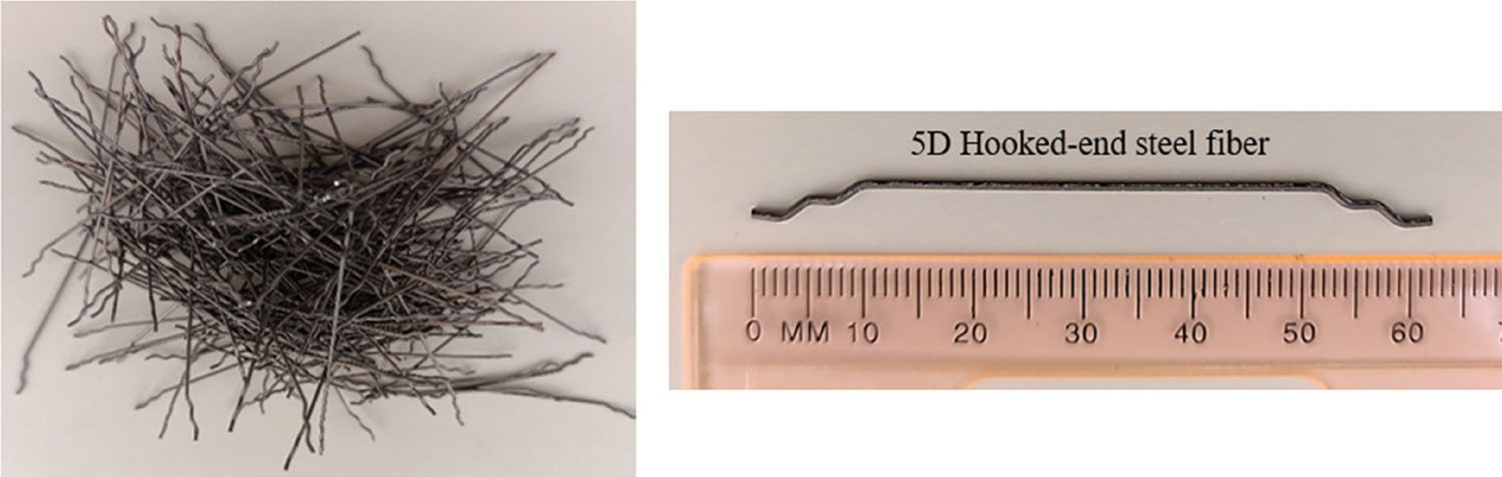
5D hooked-end steel fiber.

**Fig. 5:**
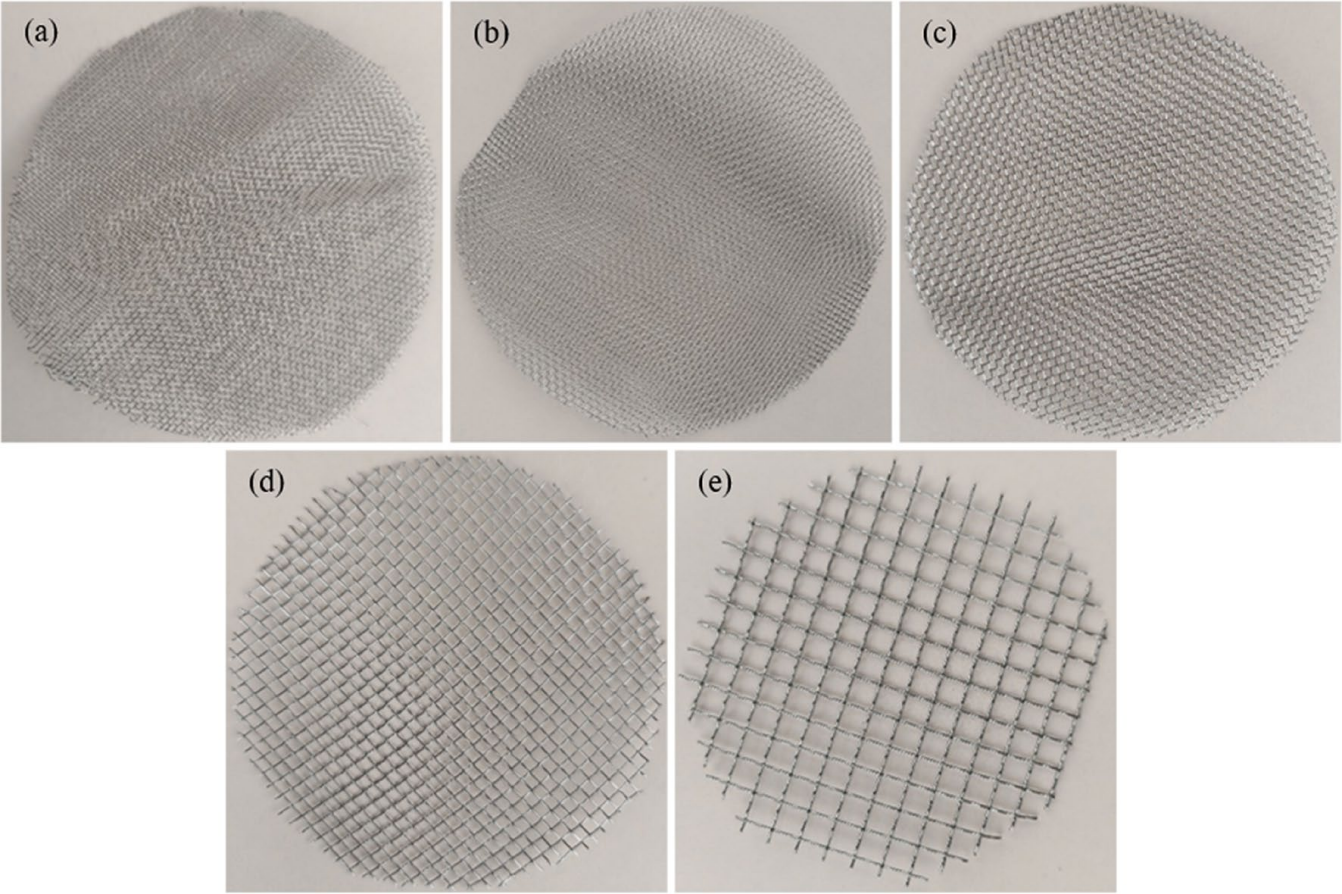
Different size of steel mesh (**a**) 40, (**b**) 30, (**c**) 20, (**d**) 10 and (**e**) 5.

**Fig. 6:**
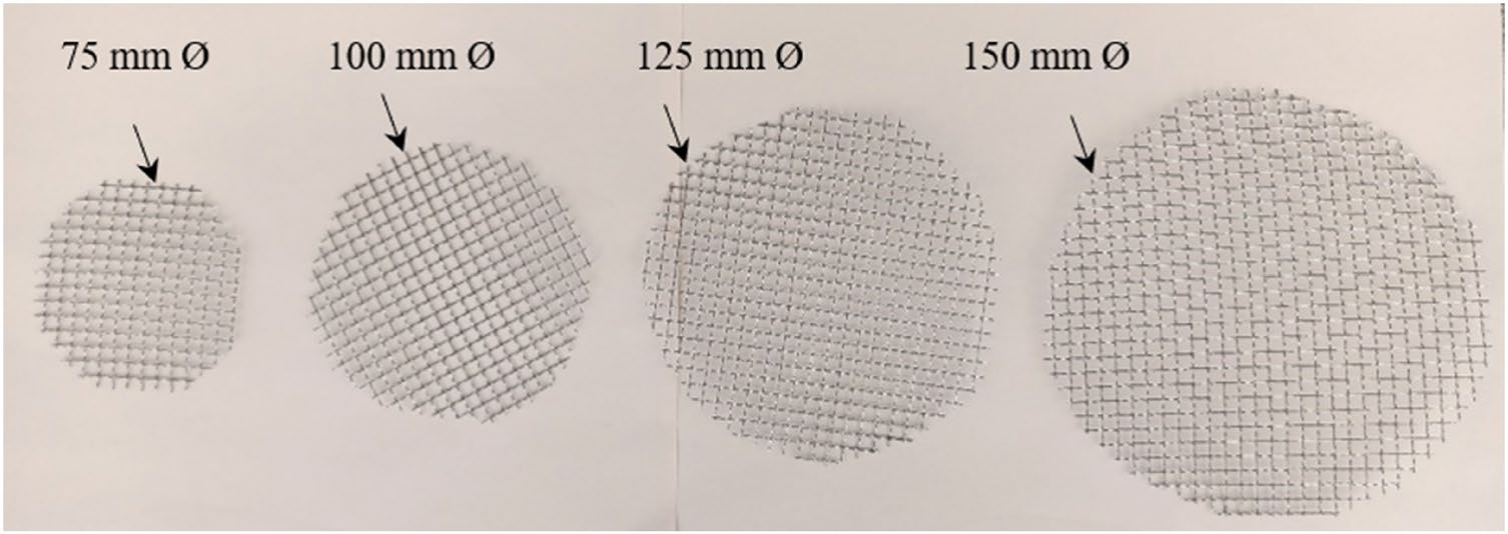
Different diameter of steel mesh of 5 type.

- The red clay is sourced from within the University Tenaga Nasional campus in Malaysia (Fig. 1). Impurities present in red clay are eliminated through a series of mechanical and manual processes. Initially, large clay agglomerates are disintegrated using manual methods. The fragmented clay is subsequently sieved through fine meshes to separate and remove gravel, stones, plant roots, and other coarse particles (Fig. 2a). In traditional or small-scale practices, visible contaminants such as organic matter, stones, and debris are manually extracted to enhance the clay’s suitability for further use. Red clay is first cleaned to remove impurities and then crushed and sieved to obtain a uniform particle size. It is subsequently calcined at 600 °C for 4 h to enhance its pozzolanic reactivity (Fig. 3). After cooling, the calcined clay is ready for incorporation into the cementitious matrix. The Class C fly ash employed in this study was sourced from the Tanjung Bin Energy Power Plant located in Johor, Malaysia, while the slag is obtained from a local supplier in Malaysia. The appearance of fly ash, slag, and red clay is shown in the Fig. 2b,c. The chemical composition of the fly ash includes 27.8% calcium oxide (CaO), which exceeds the 20% threshold, thereby supporting its classification as Class C fly ash. The particle size of the fly ash ranged from 1.88 to 76 μm, with a specific gravity of 2.27. The chemical composition of materials are shown in Table 1.
- River sand and gravel were sourced from a hardware supplier in Malaysia. The fine aggregates consisted of river sand, while gravel served as the coarse aggregate. The river sand, predominantly composed of quartz, exhibited a silica (SiO_2_) content of 96.60%. Its particle size distribution ranged from 0.07 mm to 4.75 mm, whereas the grit size spanned from 8 mm to 16 mm (Fig. 2d,e). The sodium silicate and sodium hydroxide were used as a liquid activator for producing geopolymer grout.
- The 5D hooked-end steel fibers employed in this study were characterized by Young’s modulus of 200,000 N/mm², a tensile strength of 2,300 N/mm², a length of 60 ± 2 mm, and a diameter of 0.9 mm. The appearance of the fibers is shown in Fig. 4.
- Mesh 304 stainless steel mesh of five different sizes (5, 10, 20, 30 and 40) were used (Fig. 5). The mesh sizes of 5, 10, 20, 30, and 40 correspond to the number of openings per linear inch in the mesh structure, serving as a measure to define the particle dimensions that can pass through the mesh with diameter of 0.3 to 0.6 mm. The tensile strength of the mesh is approximately 515 MPa. The mesh was fabricated into circular shapes with diameters of 75, 100, 125, and 150 mm for incorporation into PAC. A typical 5-mesh size with varying diameters is illustrated in Fig. 6.

**Table 1:** Chemical properties of materials (%).

Materials	Al_2_O_3_	SiO_2_	*P*_2_O_5_	SO_3_	K_2_O	CaO	TiO_2_	Fe_2_O_3_	MnO	MgO	Na_2_O
Red clay	35.2	43.2	0.87	0.11	1.12	0.2	3.07	12.8	0.91	0.53	1.39
Fly ash	9.75	20.4	0.83	1.85	1.09	27.8	0.63	17.2	0.23	10.2	−
Slag	13.5	27.6	0.64	1.45	0.64	33	0.51	0.43	0.66	8.93	−

### SEM and XRD analysis of raw materials

**Fig. 7:**
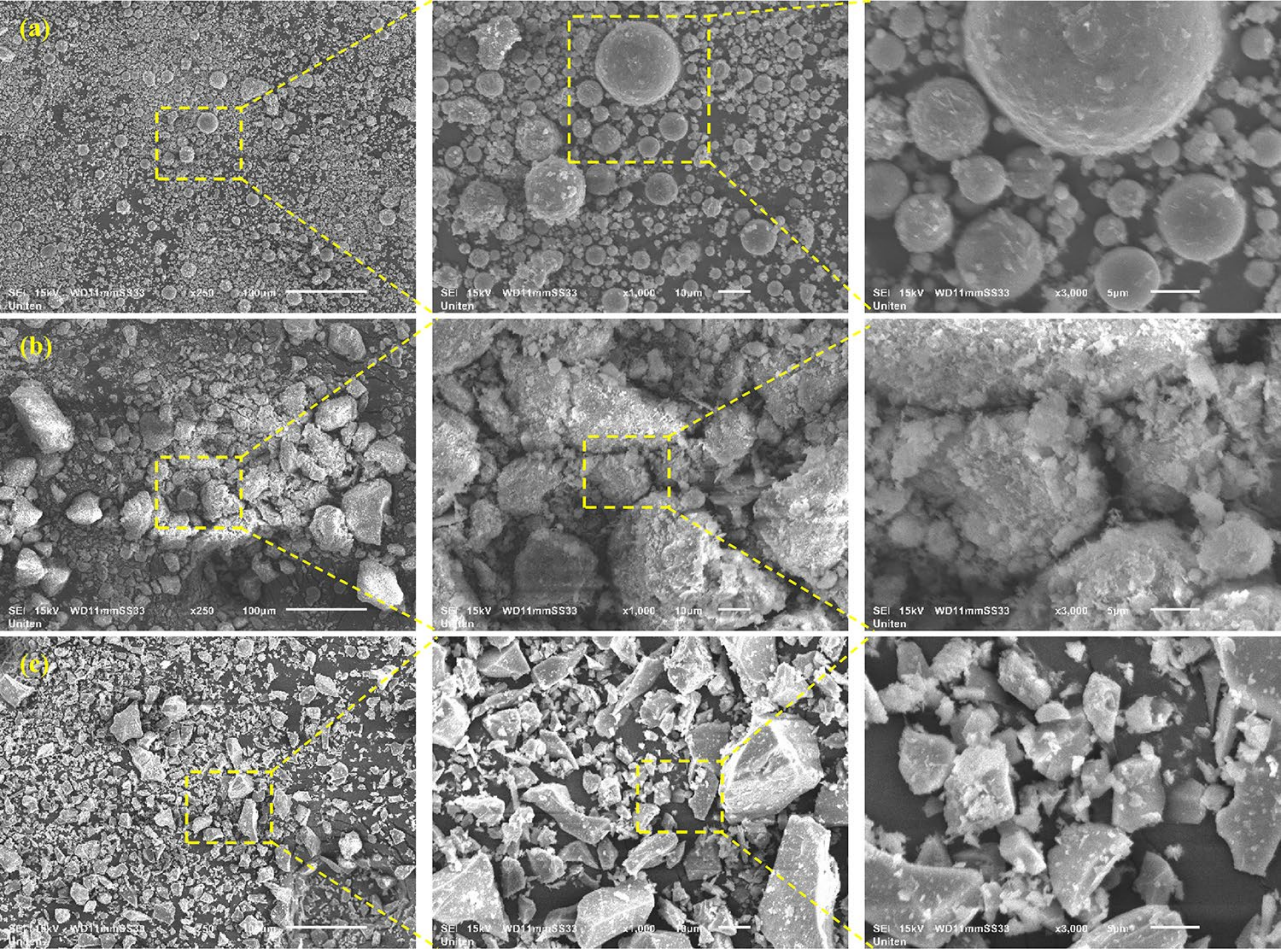
SEM analysis (**a**) FA, (**b**) red clay, and (**c**) slag.

**Fig. 8:**
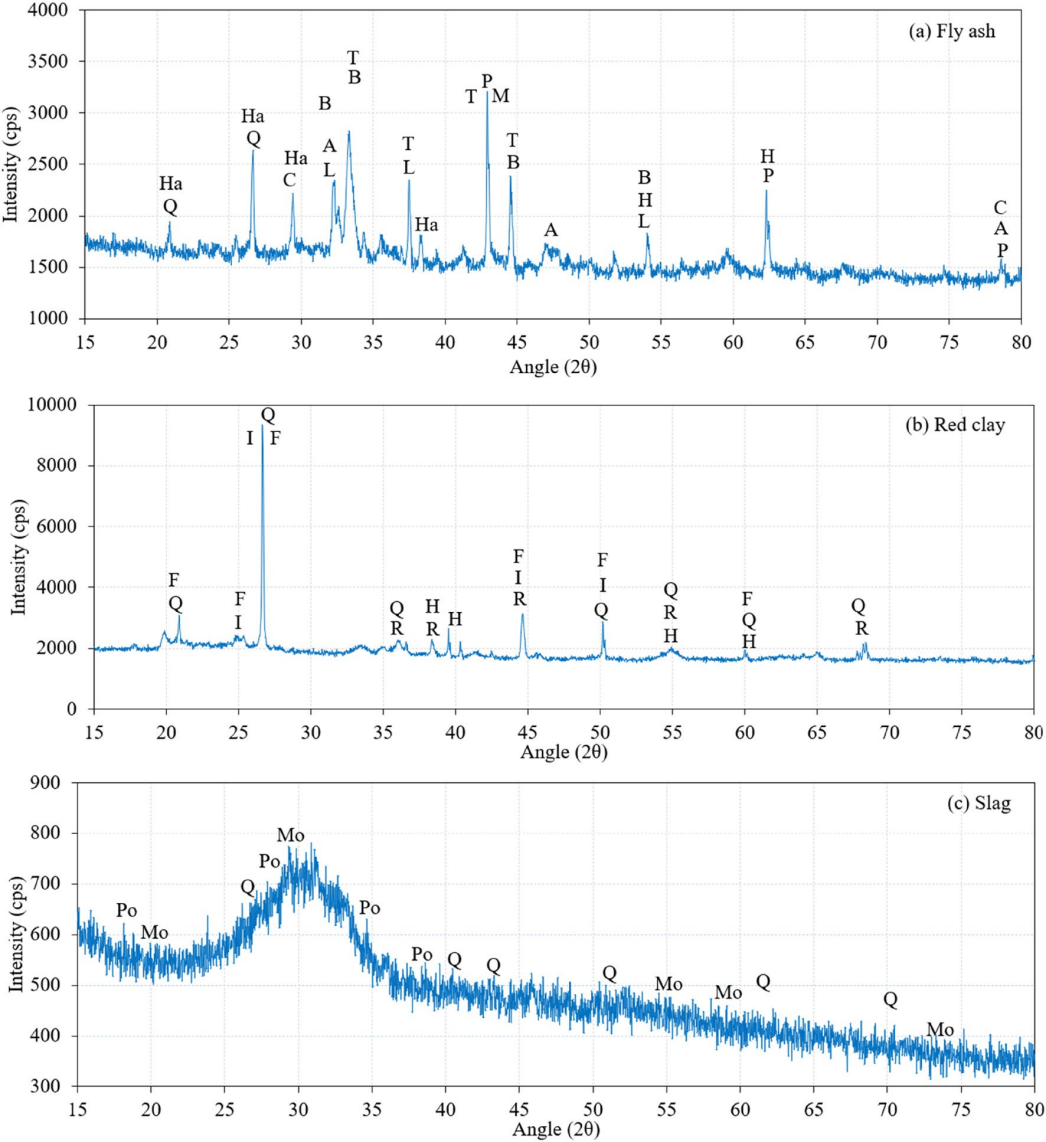
XRD analysis (**a**) Slag, (**b**) Red Clay, and (**c**) Slag (Q: Quartz, I: Illite 2M1, F: Feldspar, H: Hematite, R: Rutile, Ha: Hatrurite, C: Calcite, B: Brownmillerite, A: Anhydrite, L: Lime, M: Mullite, T: Tricalcium Dialuminate, P: Periclase, Mo: Monticellite, Po: Portlandite).

The morphological characteristics of FA, RC, and slag are illustrated in Fig. 7. Fly ash predominantly comprises spherical particles^39^, including glassy hollow cenospheres and denser plerospheres (Fig. 7a). Although most particles possess a smooth surface texture, variations in combustion parameters can lead to the formation of porous or irregular surface structures. Red clay is characterized by an angular and irregular morphology, frequently incorporating flaky particles (Fig. 7b). Its surface texture is notably rough and porous, exhibiting high reactivity due to its ultrafine particle size and substantial surface area^39^. Slag exhibits a predominantly angular and irregular morphology^39^, often displaying a coarse surface texture (Fig. 7c). Its composition is primarily amorphous and glassy, interspersed with crystalline phases. The high proportion of the amorphous phase enhances its chemical reactivity, making it highly effective in alkali-activated systems and cementitious hydration processes. Figure 8a presents the XRD analysis of fly ash, revealing the presence of Hatrurite, Mullite, Tricalcium Dialuminate, Periclase, Calcite, and Quartz as the primary crystalline phases. Hatrurite enhances early strength in cementitious materials by forming calcium silicate hydrate (C-S-H) upon hydration. Tricalcium Dialuminate reacts moderately with sulfate ions to facilitate early hydration but may cause sulfate attack in high-sulfate conditions. Calcite enhances reactivity through secondary reactions with aluminates, improving workability, although it may lower overall pozzolanic activity. Quartz acts as an inert filler with minimal contribution to hydration. Red clay exhibits peaks corresponding to Illite, Feldspar, and Quartz in its mineral composition (Fig. 8b). Illite enhances the plasticity and workability of red clay, while Feldspar minerals, including alkali feldspar and plagioclase, facilitate the formation of an amorphous glassy phase during processing. These minerals may also engage in reactions that improve the material’s reactivity and binding properties. Monticellite, a calcium magnesium silicate that crystallizes during slag solidification, enhances the pozzolanic properties of slag. This mineral increases the reactivity and binding potential in cementitious formulations, leading to enhanced mechanical properties and durability. The slag contains multiple peaks of Monticellite (Fig. 8c), which exhibits moderate pozzolanic activity, enhancing mechanical properties and durability. Additionally, Portlandite is present, contributing significantly to early strength development, alkalinity, and the formation of C-S-H.

### Mixing proportions and specimen preparation

A total of 42 distinct mixing combinations were developed and categorized into 10 groups for this study. The initial five groups comprised non-fibrous geopolymer grout-based PAC incorporating SWM at the mid height of the specimen, while the subsequent five groups included fibrous PAC designed with fibers and SWM. Among these, the first two mixtures, designated as PAC and FPAC, represented the fiber-free and fibrous specimens, respectively, serving as reference specimens for comparative analysis with the 10 groups of mixtures. The mixture identification followed a systematic nomenclature. For instance, the first group and first specimen were designated as M40-D75, where “M40” refers to the SWM type and “D75” denotes the 75 mm diameter. Similarly, fibrous specimens were denoted by adding an “F” prefix to the mixture id (e.g., F-M40-D75). The detailed composition and naming conventions for all mixing combinations are provided in Table 2.

**Fig. 9:**
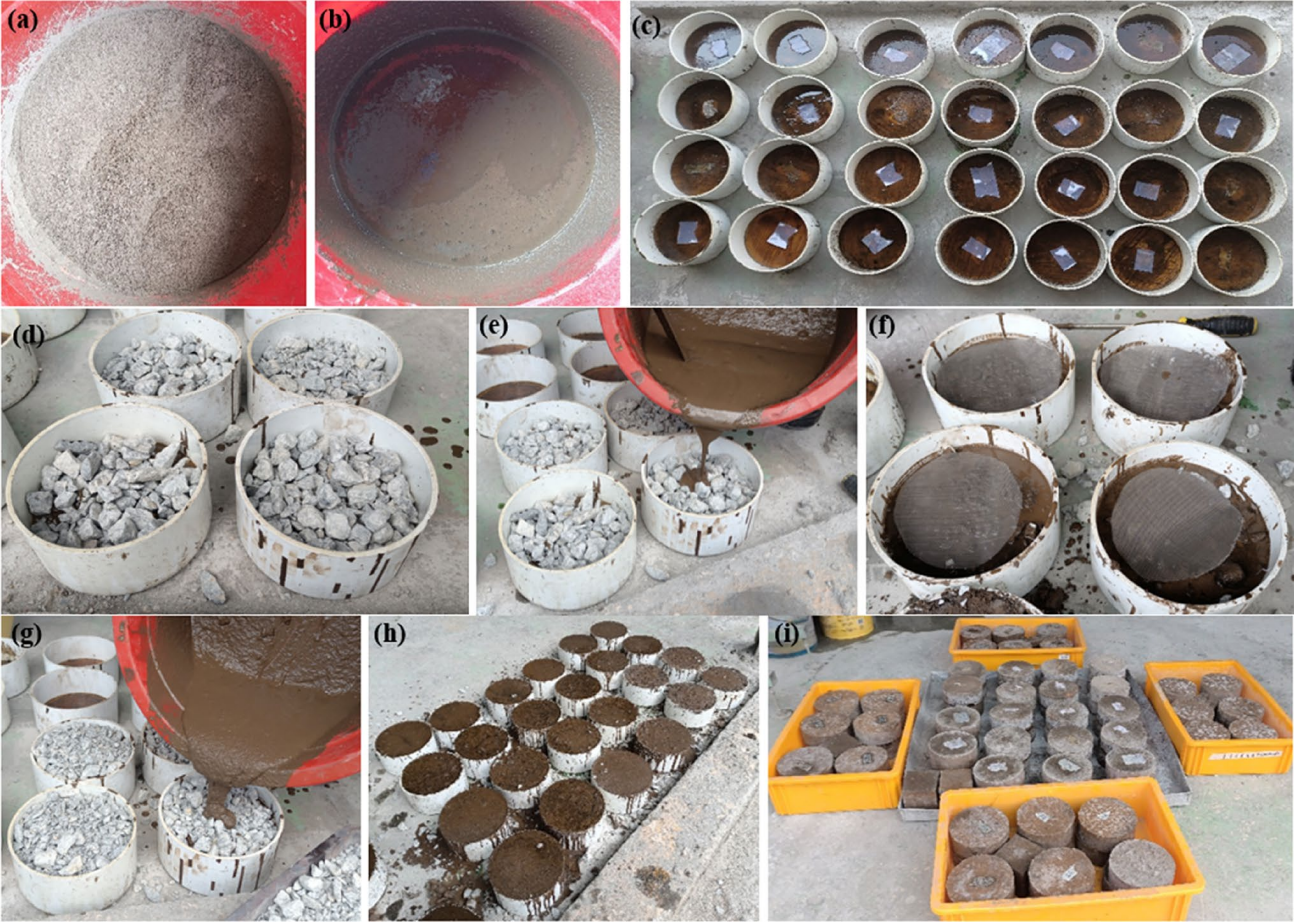
Casting procedure (**a**) dry mixture, (**b**) geopolymer grout, (**c**) empty mold, (**d**) first layer prepacked aggregates, (**e**) grouting first layer, (**f**) mesh placement, (**g**) grouting second layer, (**h**) finished specimens and (**i**) ambient curing of the specimens.

The fabrication of PAC encompassed several meticulously executed stages to ensure superior quality and performance (Fig. 9). To simplify demolding and achieve a refined surface texture, the internal surfaces of the formwork were systematically treated with a uniform application of oil, as illustrated in Fig. 9c. This treatment acted as a non-adhesive barrier, effectively mitigating the potential bonding between the formwork and the coarse aggregates during the casting process. In the subsequent step, coarse aggregates were strategically arranged within the formwork to construct a stable foundational framework for the initial PAC layer, as shown in Fig. 9d. For fibrous PAC, aggregates were homogeneously mixed with fibers before being compactly positioned within the mold. Thereafter, a geopolymer grout was uniformly introduced onto the surface of the preliminary PAC layer. The grout infiltrated the voids and interstices among the aggregates and fibers, as depicted in Fig. 9e. The next phase involved placing a circular SWM at the top of the first layer, as demonstrated in Fig. 9f, followed by sequentially adding aggregates and fibers. The process was finalized with the grouting of the second layer, as shown in Fig. 9g. The completed specimen’s external appearance is presented in Fig. 9h. All specimens were subjected to ambient curing conditions for a duration of 28 days, as indicated in Fig. 9i. It is essential to emphasize that the casting process must be completed within 5 min to account for the rapid setting time of the geopolymer grout due to the high reactivity of its alkaline activators with the aluminosilicate materials. In the PAC method, the aggregates and fibers are arranged randomly within the formwork, followed by the systematic injection of grout. A crucial aspect of this process involved light compaction using a < 6 mm diameter steel rod to ensure the grout effectively filled any voids and achieved optimal densification.

**Table 2:** Mix proportions.

Group	Mix id	Binder/sand	Fly ash (%)	Slag (%)	Red clay (%)	w/b	Mesh size	Mesh diameter	Fiber (%)	SP
1	PAC	1.0	40	40	20	0.5	0	0	0	0.8
	FPAC	1.0	40	40	20	0.5	0	0	2.5	1.0
	M40-D75	1.0	40	40	20	0.5	40	75	0	0.8
	M40-D100	1.0	40	40	20	0.5		100	0	0.8
	M40-D125	1.0	40	40	20	0.5		125	0	0.8
	M40-D150	1.0	40	40	20	0.5		150	0	0.8
2	M30-D75	1.0	40	40	20	0.5	30	75	0	0.8
	M30-D100	1.0	40	40	20	0.5		100	0	0.8
	M30-D125	1.0	40	40	20	0.5		125	0	0.8
	M30-D150	1.0	40	40	20	0.5		150	0	0.8
3	M20-D75	1.0	40	40	20	0.5	20	75	0	0.8
	M20-D100	1.0	40	40	20	0.5		100	0	0.8
	M20-D125	1.0	40	40	20	0.5		125	0	0.8
	M20-D150	1.0	40	40	20	0.5		150	0	0.8
4	M10-D75	1.0	40	40	20	0.5	10	75	0	0.8
	M10-D100	1.0	40	40	20	0.5		100	0	0.8
	M10-D125	1.0	40	40	20	0.5		125	0	0.8
	M10-D150	1.0	40	40	20	0.5		150	0	0.8
5	M5-D75	1.0	40	40	20	0.5	5	75	0	0.8
	M5-D100	1.0	40	40	20	0.5		100	0	0.8
	M5-D125	1.0	40	40	20	0.5		125	0	0.8
	M5-D150	1.0	40	40	20	0.5		150	0	0.8
6	F-M40-D75	1.0	40	40	20	0.5	40	75	2.5	1.0
	F-M40-D100	1.0	40	40	20	0.5		100	2.5	1.0
	F-M40-D125	1.0	40	40	20	0.5		125	2.5	1.0
	F-M40-D150	1.0	40	40	20	0.5		150	2.5	1.0
7	F-M30-D75	1.0	40	40	20	0.5	30	75	2.5	1.0
	F-M30-D100	1.0	40	40	20	0.5		100	2.5	1.0
	F-M30-D125	1.0	40	40	20	0.5		125	2.5	1.0
	F-M30-D150	1.0	40	40	20	0.5		150	2.5	1.0
8	F-M20-D75	1.0	40	40	20	0.5	20	75	2.5	1.0
	F-M20-D100	1.0	40	40	20	0.5		100	2.5	1.0
	F-M20-D125	1.0	40	40	20	0.5		125	2.5	1.0
	F-M20-D150	1.0	40	40	20	0.5		150	2.5	1.0
9	F-M10-D75	1.0	40	40	20	0.5	10	75	2.5	1.0
	F-M10-D100	1.0	40	40	20	0.5		100	2.5	1.0
	F-M10-D125	1.0	40	40	20	0.5		125	2.5	1.0
	F-M10-D150	1.0	40	40	20	0.5		150	2.5	1.0
10	F-M5-D75	1.0	40	40	20	0.5	5	75	2.5	1.0
	F-M5-D100	1.0	40	40	20	0.5		100	2.5	1.0
	F-M5-D125	1.0	40	40	20	0.5		125	2.5	1.0
	F-M5-D150	1.0	40	40	20	0.5		150	2.5	1.0

w/b: water to binder ratio, SP: superplasticizer, M5-M40: mesh type used, D75-15: diameter of steel wire mesh.

### Impact test setup

**Fig. 10:**
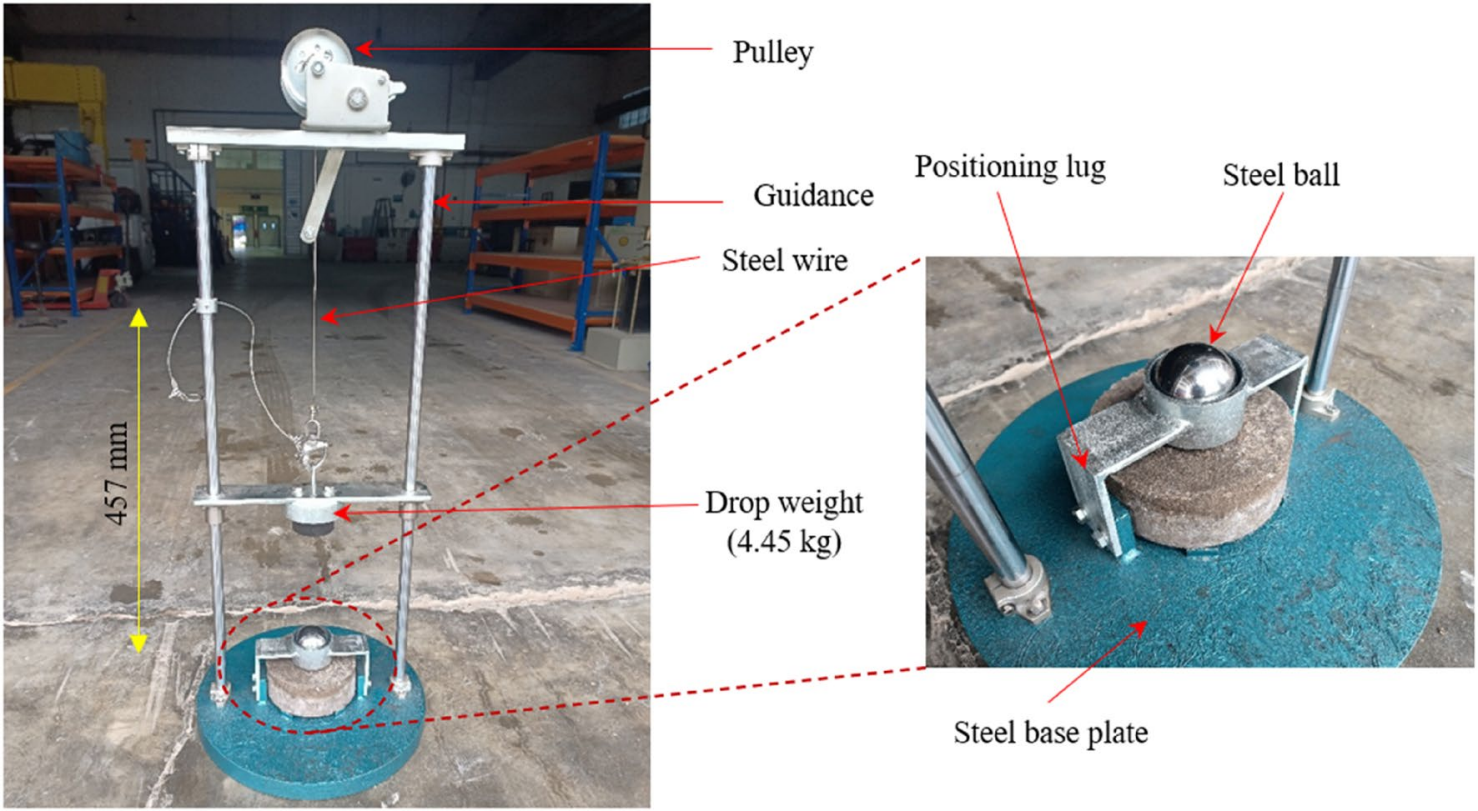
Impact testing device.

The impact strength testing procedure in this study was grounded in the well-established procedures set forth by ACI Committee 544^42^, utilizing a method known for its practicality, precision, and cost-effectiveness the drop-weight test. This approach measures the energy absorbed by the specimen by counting the number of impacts necessary to reach a predefined damage threshold, which includes both the formation of the initial crack (G1) and the final failure point (G2). Notably, this impact test is streamlined and focused, quantifying only the number of impacts while deliberately excluding other variables such as load-time history, vibration, and strain. As stipulated by ACI 544^42^, the test is versatile, enabling the comparison of impact strengths across various concrete types, regardless of their thickness. In the present experiment, a cylindrical disc with a thickness of 63.5 mm and a diameter of 150 mm was subjected to a series of repeated impacts, as shown in Figure. 10. A 4.45 kg weight, dropped from a height of 0.457 m, delivered these impacts. The objective was to observe the disc’s behavior under repeated impact forces, following a methodology frequently used in previous studies^10,31,43^. The resulting data were meticulously analyzed, with particular attention paid to the W1 and W2 parameters. Based on the experimental data collected, the impact energy (U) of the specimens was calculated using Eq. (1).

1 $$\:U=m\times\:g\times\:h\times\:G$$

Where: *g*: Gravitational acceleration, *G*: number of impacts, *m*: mass (4.45 kg) of the dropped object and *h*: height from which the mass is released.

## Findings and analysis

To evaluate compressive strength, 100 mm cube specimens were prepared in accordance with the provisions outlined in IS:516^44^. The experimental analysis was conducted using the average values obtained from three replicate specimens for both compressive and impact strength. The fiber-free specimens exhibited a compressive strength of 38.52 MPa. This strength is primarily attributed to the red clay and fly ash-based geopolymers, which serve as the main sources of silica and alumina, though with limited reactivity. The incorporation of slag contributes additional reactive silica and alumina, while also supplying calcium and facilitating alkali extraction factors that enhance both dissolution and polymerization processes 33. In contrast, the fibrous specimens achieved a significantly higher compressive strength of 52.65 MPa, demonstrating the effectiveness of 5D steel fiber reinforcement in improving compressive performance. Adding 2.5% of 5D steel fibers to this specimen exhibited a superior compressive strength of 36.68% higher than the fiber-free specimens. This phenomenon is due to each fiber serves as a micro-scale energy dissipator that spans across cracks, contributing to an efficient stress transfer mechanism^45^. This mechanism reduces the stress concentration at the crack tip, preventing brittle failure and enhancing the material’s ductility, resulting in an enhancement of compressive strength. The presence of steel fibers significantly enhances compressive strength, attributed to their high tensile strength and the increased energy required to deform the hooked ends of the fibers^46^. The improvement in compressive strength of geopolymer PAC with fiber addition is due to the synergistic interaction between steel fibers and the matrix. Beyond their tensile strength, steel fibers bridge and arrest microcracks during early loading, delaying crack propagation and failure. Their mechanical interlock and frictional bond with the matrix enhance stress redistribution and resist pull-out under compression, resulting in improved strength, ductility, and a more cohesive failure mode. This notable increase in compressive strength proves the combined impact of slag-induced geopolymerization and fiber reinforcement, where 5D steel fibers enhance load distribution by bridging cracks and absorbing energy, efficiently decrease stress development and enhancing the overall mechanical performance of the concrete. The impact strength results of all mixtures are presented in Table 3.

**Table 3:** Findings of the impact strength.

Mixture id	Specimen 1		Specimen 2		Specimen 3		Mean		SD		COV	
	G1	G2	G1	G2	G1	G2	G1	G2	G1	G2	G1	G2
PAC	25	28	24	26	21	25	23	26	2.08	1.53	8.92	5.80
FPAC	81	315	92	356	85	329	86	333	5.57	20.84	6.47	6.25
M40-D75	24	32	26	34	25	29	25	32	1.00	2.52	4.00	7.95
M40-D100	26	37	27	39	25	40	26	39	1.00	1.53	3.85	3.95
M40-D125	25	39	25	49	27	44	26	44	1.15	5.00	4.50	11.36
M40-D150	26	56	28	49	26	65	27	57	1.15	8.02	4.33	14.15
M30-D75	25	35	27	39	30	37	27	37	2.52	2.00	9.21	5.41
M30-D100	26	40	27	53	28	49	27	47	1.00	6.66	3.70	14.07
M30-D125	24	50	29	45	32	58	28	51	4.04	6.56	14.26	12.86
M30-D150	30	63	31	60	27	72	29	65	2.08	6.24	7.10	9.61
M20-D75	26	38	27	45	28	56	27	46	1.00	9.07	3.70	19.58
M20-D100	29	55	29	40	27	59	28	51	1.15	10.02	4.08	19.51
M20-D125	31	49	27	60	30	68	29	59	2.08	9.54	7.10	16.17
M20-D150	32	68	27	78	29	71	29	72	2.52	5.13	8.58	7.09
M10-D75	26	50	29	42	28	61	28	51	1.53	9.54	5.52	18.70
M10-D100	25	65	27	54	29	50	27	56	2.00	7.77	7.41	13.79
M10-D125	26	52	27	61	30	75	28	63	2.08	11.59	7.52	18.50
M10-D150	29	81	28	70	33	65	30	72	2.65	8.19	8.82	11.37
M5-D75	28	52	31	60	27	72	29	61	2.08	10.07	7.26	16.41
M5-D100	29	65	29	79	31	72	30	72	1.15	7.00	3.89	9.72
M5-D125	30	81	31	75	32	77	31	78	1.00	3.06	3.23	3.93
M5-D150	29	79	32	98	30	85	30	87	1.53	9.71	5.04	11.12
F-M40-D75	88	330	89	356	93	375	90	354	2.65	22.59	2.94	6.39
F-M40-D100	87	346	92	369	93	386	91	367	3.21	20.07	3.55	5.47
F-M40-D125	92	350	85	395	93	384	90	376	4.36	23.46	4.84	6.23
F-M40-D150	87	401	100	376	96	406	94	394	6.66	16.07	7.06	4.08
F-M30-D75	89	345	90	362	94	381	91	363	2.65	18.01	2.91	4.97
F-M30-D100	88	350	92	390	89	372	90	371	2.08	20.03	2.32	5.40
F-M30-D125	90	399	88	384	91	375	90	386	1.53	12.12	1.70	3.14
F-M30-D150	92	395	86	408	94	436	91	413	4.16	20.95	4.59	5.07
F-M20-D75	85	385	98	404	94	370	92	386	6.66	17.04	7.21	4.41
F-M20-D100	88	415	92	399	94	412	91	409	3.06	8.50	3.34	2.08
F-M20-D125	87	444	91	410	98	425	92	426	5.57	17.04	6.05	4.00
F-M20-D150	95	470	90	455	101	420	95	448	5.51	25.66	5.78	5.72
F-M10-D75	101	420	98	436	88	401	96	419	6.81	17.52	7.12	4.18
F-M10-D100	83	460	112	433	100	421	98	438	14.57	19.97	14.82	4.56
F-M10-D125	95	439	98	480	102	440	98	453	3.51	23.39	3.57	5.16
F-M10-D150	102	467	94	444	97	497	98	469	4.04	26.58	4.14	5.66
F-M5-D75	90	465	108	435	99	406	99	435	9.00	29.50	9.09	6.78
F-M5-D100	101	475	101	462	93	429	98	455	4.62	23.71	4.70	5.21
F-M5-D125	97	454	95	482	100	468	97	468	2.52	14.00	2.59	2.99
F-M5-D150	89	501	102	483	106	468	99	484	8.89	16.52	8.98	3.41

COV: coefficient of variation, SD: standard deviation,

### Effect of M40-type SWM and fibers on the impact strength

**Fig. 11:**
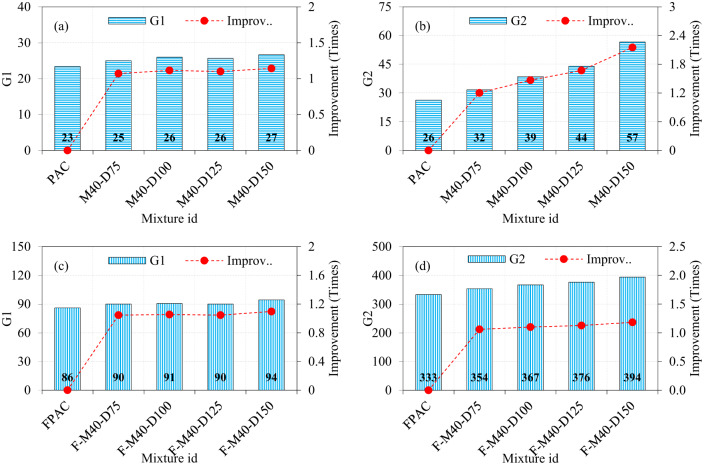
The effect of incorporating M40-type SWM and fibers on impact strength of PAC.

The results presented in the figure underscore the individual and combined contributions of steel wire mesh (SWM) and steel fibers to the impact strength of PAC. The baseline G1 and G2 values of the PAC specimens were recorded as 23 and 26, respectively, establishing reference points for evaluating various reinforcement configurations. Introducing M40-type SWM with diameters of 75, 100, 125, and 150 mm to PAC resulted in G1 values of 25, 26, 26, and 27, respectively (Fig. 11a). This indicates marginal improvements of 1.07, 1.11, 1.11, and 1.14 times compared to the control PAC specimen. The modest improvement in G1 suggests that the placement of SWM at the mid-depth primarily resists tensile stresses caused by impact loads, but does not significantly enhance the G1 due to limited interaction with the surrounding matrix. In contrast, the G2 values for the same specimens showed a more pronounced enhancement, recording values of 32, 39, 44, and 57, corresponding to improvements of 1.20, 1.46, 1.67, and 2.15 times, respectively (Fig. 11b). This significant increase in G2 highlights the critical role of SWM in delaying crack propagation and structural failure. The disparity between G1 and G2 enhancements underscores the SWM’s greater efficacy in post-cracking behavior rather than initial crack resistance. The findings of this research are consistent with those reported in previous studies^10^. The progressive increase in G2 with larger SWM diameters also suggests that greater coverage and distribution of tensile forces contribute to improved energy dissipation under impact loads^10^.

Fibrous PAC specimens incorporating both SWM and steel fibers demonstrated substantial improvements in both G1 and G2. The G1 values for specimens with M40-type SWM of diameters 75, 100, 125, and 150 mm were 90, 91, 90, and 94, respectively, reflecting enhancements of 1.04, 1.05, 1.04, and 1.09 times compared to PAC specimens (Fig. 11c). The consistent G1 values across varying SWM diameters suggest that the synergistic action of steel fibers and SWM ensures a uniformly enhanced initial crack resistance. Remarkably, the G2 values for these specimens exhibited significant improvements, with values of 354, 367, 376, and 394 for SWM diameters of 75, 100, 125, and 150 mm, corresponding to improvements of 1.06, 1.10, 1.13, and 1.18 times over PAC specimens (Fig. 11d). These findings indicate that the combined reinforcement creates a highly effective multi-scale reinforcement mechanism. The SWM inhibits the formation and widening of macro-cracks, while steel fibers regulate micro-cracking, enhance the bond with the matrix^45^, and mitigate spalling. This dual mechanism results in superior energy absorption capacity and impact resistance.

### Effect of M30-type SWM and fibers on the impact strength

**Fig. 12:**
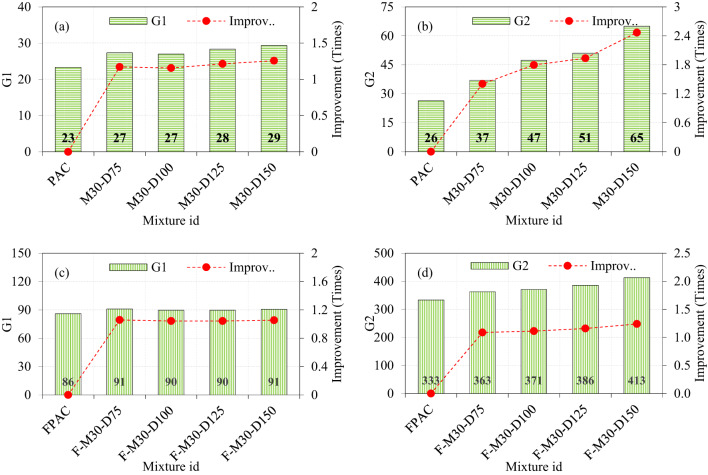
The effect of incorporating M30-type SWM and fibers on impact strength of PAC.

The introduction of M30-type SWM with diameters of 75, 100, 125, and 150 mm into PAC resulted in G1 values of approximately 27, 27, 28, and 29, respectively. These values reflect increases of 1.17, 1.15, 1.21, and 1.25 times compared to the control PAC specimens (Fig. 12a). The marginal variation in G1 across different diameters suggests that the influence of SWM diameter on G1 is limited, likely due to its placement at the mid-depth of the specimens, which minimizes its impact on stress distribution. In contrast, the G2 values for M30-type SWM show a clear dependency on diameter, with recorded values of 37, 47, 51, and 65 for diameters of 75, 100, 125, and 150 mm, respectively (Fig. 12b). These correspond to significant improvements of 1.40, 1.80, 1.94, and 2.47 times over the control PAC specimens. The pronounced enhancement in G2 with increasing diameter highlights the critical role of SWM size in improving energy dissipation and load-bearing capacity. Among the tested diameters, the 150 mm SWM delivered the best performance in G2, indicating its superiority in enhancing structural behavior. The SWM significantly enhances the energy absorption capacity of the composite by effectively dispersing the impact energy over a larger area and extended duration. This distribution helps reduce the intensity of stress concentration at localized points, thereby minimizing the likelihood of crack initiation and propagation^10^. As a result, the overall structural integrity of the PAC is improved, making it more resilient to impact loading conditions.

The fibrous PAC specimens exhibited a synergistic effect between steel fibers and SWM, resulting in higher G1 and G2 values. For M30-type SWM with diameters of 75, 100, 125, and 150 mm, the G1 values were recorded as 91, 90, 90, and 91, representing slight improvements of 1.06, 1.04, 1.04, and 1.05 times compared to the control PAC specimens (Fig. 12c). This limited increase in G1 suggests that the uniform stress distribution provided by the steel fibers reduces the sensitivity of G1 to SWM diameter variations. The G2 values for fibrous PAC specimens, however, were remarkable. For M30-type SWM, the recorded G2 values were 363, 371, 386, and 413 for diameters of 75, 100, 125, and 150 mm, respectively, corresponding to improvements of 1.08, 1.11, 1.16, and 1.24 times over the control PAC specimens (Fig. 12d). The consistent enhancement in G2 with increasing diameter, coupled with the synergistic effect of fibers, indicates that the combination of larger SWM diameters and steel fibers is particularly effective in optimizing energy absorption and structural resilience. Notably, the 150 mm diameter SWM again demonstrated the best performance, cementing its role as the optimal choice for maximizing both G1 and G2 in fibrous PAC systems. The hooked ends of the fibers facilitate mechanical interlocking within the concrete matrix^47^, while the SWM provides supplementary reinforcement to optimize the functionality of the fibers. This interaction ensures efficient stress transfer from the matrix to the fibers, thereby enhancing the overall toughness of the PAC^48^. Additionally, the combined presence of fibers and SWM impedes crack propagation by introducing additional resistance along the crack path. Rather than experiencing brittle fracture, the fibers and SWM undergo pullout, which results in energy dissipation and delays the onset of failure.

When the diameter of the SWM is increased, and the G2 values are observed to increase and the same behavior recorded for the M-40 type mesh as well, several factors can explain this behavior: (i) The increase in SWM diameters enhances the surface area and volume available for energy absorption during impact. This leads to a more effective distribution of impact energy across the material, reducing localized stress concentrations and preventing the formation of cracks^49^. As a result, the composite demonstrates improved energy absorption capacity before failure, thereby enhancing its overall impact resistance. (ii) Larger SWM sizes improve the mesh’s ability to distribute the applied load over a wider area. This increased load distribution reduces the intensity of localized strains, thereby decreasing the likelihood of material failure at specific points. (iii) The incorporation of larger SWM components fosters more effective interactions with the surrounding PAC matrix, thereby improving adhesion and overall structural integrity. Enhanced bonding between the SWM and the matrix facilitates superior energy transfer and absorption, which contributes to an increase in impact strength. (iv) Larger SWM diameters can impede the rapid progression of cracks by providing a more robust barrier against crack propagation. The increased dimensions of the mesh allow it to bridge wider fissures, thereby preventing their swift growth and enhancing the PAC’s overall toughness^31^. (v) In PAC, the integration of larger SWM with additional constituents, such as fibers or aggregates, can induce synergistic effects that further enhance impact strength. The increased diameter of the SWM works in conjunction with these components, leading to improved performance under dynamic loading conditions. The enhanced impact resistance reveals how increasing the SWM diameter strengthens PAC by enhancing energy absorption, distributing loads more effectively, improving adhesion with the matrix, and slowing crack propagation, resulting in a more durable and resilient material under dynamic forces.

### Effect of M20-type SWM and fibers on the impact strength

**Fig. 13:**
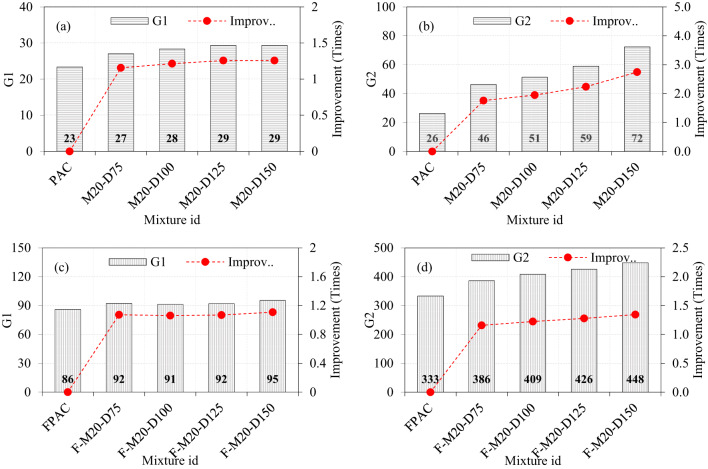
The effect of incorporating M20-type SWM and fibers on the impact strength of PAC.

Figure 13 illustrates the influence of increasing the diameter of M20-type SWM in PAC on the properties G1 and G2. A marginal enhancement in G1 and a pronounced improvement in G2 were observed. Specifically, incorporating a 75 mm diameter SWM led to approximately 1.16 and 1.75 times increases in G1 and G2, respectively, compared to PAC without SWM. Expanding the SWM diameter to 100 mm further amplified the G1 and G2 values by 1.21 and 1.95 times, respectively. Notably, G1 exhibited a continued incremental improvement of about 1.26 times with 125 mm and 150 mm SWM diameters, whereas G2 experienced a more substantial increase, reaching 2.24 and 2.75 times higher values than the control PAC. The disparity between the improvements in G1 and G2 underscores the varying sensitivity of these parameters to SWM diameter. The substantial increase in G2 likely reflects the enhanced crack resistance and energy absorption capabilities provided by the larger SWM diameter, which offers better load redistribution and confinement^50^. Conversely, the modest gains in G1 suggest a limitation in the mesh’s ability to improve properties such as initial stiffness or load-bearing capacity, potentially constrained by the matrix’s inherent characteristics and bond development. The synergistic interaction between SWM and fibers in PAC specimens demonstrated a notable enhancement in both G1 and G2 values. Specifically, the G1 values ranged from 86 to 95 as the SWM diameter increased from 75 to 150 mm, while the corresponding G2 values varied between 386 and 448. When compared to conventional PAC specimens, the G1 values exhibited incremental improvements of 1.07, 1.06, 1.07, and 1.11 times for SWM diameters of 75, 100, 125, and 150 mm, respectively. Similarly, the G2 values increased by 1.16, 1.23, 1.28, and 1.34 times for the same SWM diameters. This trend highlights a consistent and progressive improvement in both G1 and G2 values with increasing SWM diameter, underscoring the effectiveness of larger SWM diameters in enhancing these parameters. Moreover, the pattern of improvement for G1 and G2 aligns closely with other types of SWM, suggesting a universal trend in their synergistic interaction with fibers. Notably, the greater enhancement observed in G2 values compared to G1 indicates that the combined effect of SWM and fibers may have a more pronounced impact on parameters associated with G2.

### Effect of M10-type SWM and fibers on the impact strength

**Fig. 14:**
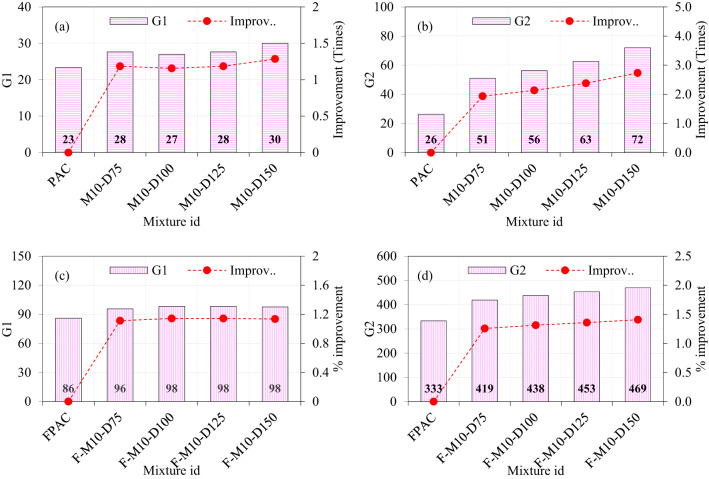
The effect of incorporating M10-type SWM and fibers on impact strength of PAC.

The integration of a 75 mm diameter of M10-type SWM demonstrated a notable performance improvement (Fig. 14), with G1 and G2 values increasing by approximately 1.19 and 1.94 times, respectively, compared to PAC without SWM (Fig. 14a, b). This initial enhancement highlights the efficacy of SWM in augmenting the structural properties of PAC. However, expanding the SWM diameter to 100 mm yielded only modest additional gains, with G1 and G2 rising by 1.14 and 2.14 times, respectively, relative to the smaller SWM. This suggests that while increasing SWM diameter positively impacts performance, the rate of improvement diminishes, particularly for G1. Further enlargement to a 125 mm SWM diameter maintained the upward trend, with G1 and G2 improving by 1.18 and 2.38 times, respectively. Notably, at this stage, G2 exhibited a more substantial enhancement, reaching 2.73 times the control PAC, whereas G1 achieved a comparatively moderate increase of 1.29 times. This disparity indicates that G2 benefits more significantly from larger SWM diameters, potentially due to factors such as enhanced stress redistribution or improved interfacial bonding mechanisms^10^. Overall, while increasing SWM diameter consistently improves G1 and G2, the diminishing incremental gains for G1 at larger diameters raise questions about the optimal balance between SWM size, material usage, and performance benefits. This suggests the need for targeted strategies to maximize efficiency without over-reliance on increasing SWM dimensions.

The synergistic interplay between M10-type SWM and fibers in PAC specimens revealed a marked improvement in both G1 and G2 values, underscoring the combined benefits of these enhancements. As the SWM diameter increased from 75 to 150 mm, G1 values exhibited a modest rise from 96 to 98, while G2 values showed a more pronounced increase, ranging from 419 to 469. These trends highlight the differential sensitivity of G2 to the SWM diameter compared to G1, suggesting distinct underlying mechanisms driving these improvements. When compared to conventional PAC specimens, G1 values demonstrated incremental improvements of 1.11, 1.14, 1.14, and 1.14 times for SWM diameters of 75, 100, 125, and 150 mm, respectively (Fig. 14c). In contrast, G2 values exhibited more significant enhancements, increasing by 1.31, 1.37, 1.40, and 1.45 times for the same SWM diameters (Fig. 14d). The consistent rise in G2 highlights its greater responsiveness to increasing SWM diameter, likely driven by improved energy dissipation and crack-bridging mechanisms facilitated by the SWM-fiber interaction^51^.

### Effect of M5-type SWM and fibers on the impact strength

**Fig. 15:**
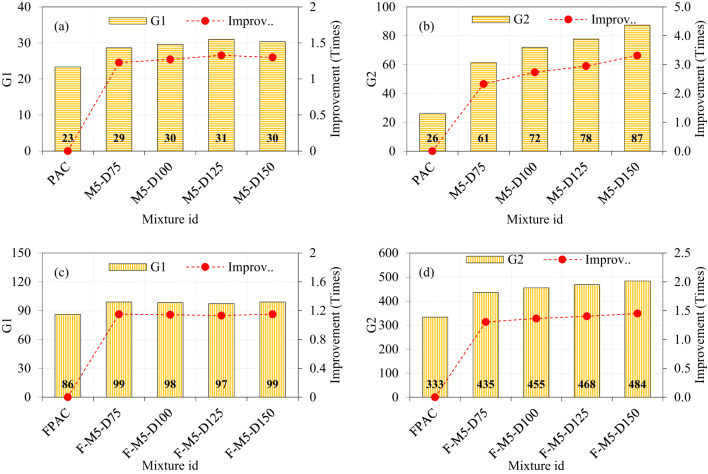
The effect of incorporating M5-type SWM and fibers on impact strength of PAC.

The performance of the M5-type SWM surpassed that of other SWM types, as evidenced by G1 values ranging from 29 to 31 for fiber-free specimens and from 97 to 99 for fibrous specimens (Fig. 15a and c). Similarly, G2 values were observed to range from 61 to 87 in fiber-free specimens and from 435 to 484 in fibrous specimens, highlighting the significant enhancement provided by fiber reinforcement (Fig. 15b and d). A marginal improvement was observed in the G1 values, whereas a significant enhancement was noted in the G2 values. Among the fiber-free and fibrous specimens, the highest G1 and G2 values were achieved by specimens with a 150 mm diameter of M5-type SWM. The observed improvements in G1 and G2 were 1.3 and 3.31, respectively, for the fiber-free specimens, and 1.15 and 1.42 times higher for the fibrous specimens, relative to the reference specimens (PAC and FPAC). The G1 value for the fiber-free specimens with a 75–125 mm diameter of M5-type SWM showed a modest improvement, ranging from 1.23 to 1.33 times higher than the PAC specimens. This indicates a consistent but relatively small enhancement in the performance of the fiber-free specimens, suggesting that the inclusion of M5-type SWM can offer incremental improvements in certain aspects of the material’s properties. In contrast, the G2 values for these specimens exhibited a more pronounced improvement, ranging from 1.32 to 1.40 times higher than the PAC specimens, highlighting a significant impact of M5-type SWM on the PAC’s impact resistance.

For the fibrous specimens with the same 75–125 mm diameter of M5-type SWM, the G1 value showed a smaller range of improvement, from 1.13 to 1.15 times compared to the FPAC specimen. This suggests that the fiber reinforcement provides a more consistent, though less dramatic, enhancement in the initial performance compared to the fiber-free variants. However, the G2 values for the fibrous specimens were significantly higher, ranging from 1.30 to 1.40 times greater than the FPAC specimen, which indicates a more substantial improvement in impact strength performance when fibers are incorporated. These results demonstrate that the inclusion of M5-type SWM leads to varying degrees of improvement in both fiber-free and fibrous specimens, with more substantial gains in G2 than in G1. Furthermore, the fibrous specimens exhibit a greater overall enhancement in G2 compared to their fiber-free counterparts, emphasizing the synergistic effect of fibers in improving the material’s mechanical and durability properties^52^.

### Comparison of SWM type and fibers on the impact strength

**Fig. 16:**
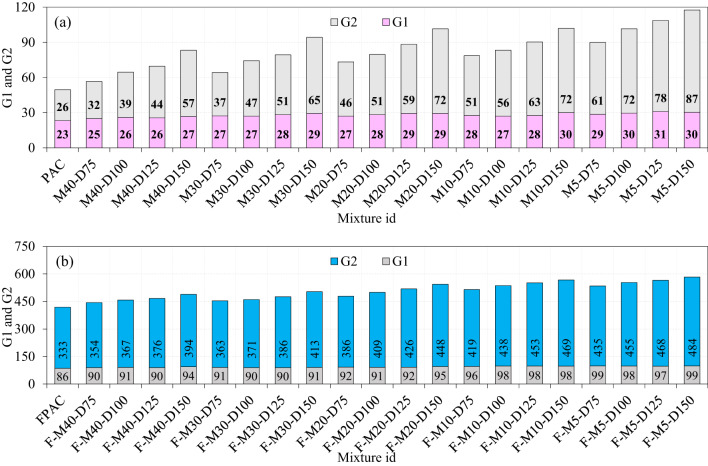
Comparison of G1 and G2 with all types of SWM and fibers.

Figure 16 presents a comprehensive comparison of the impact strength of specimens incorporating various sizes and diameters of SWM and fibers. Figure 16a highlights that the G1 values for specimens, irrespective of mesh type or diameter, ranged narrowly between 25 and 31. This marginal improvement indicates that SWM alone has limited effectiveness in resisting crack initiation. In contrast, the G2 values demonstrate a significant enhancement, with M40-type SWM ranging from 32 to 57, M30-type SWM from 37 to 65, M20-type SWM from 46 to 72, M10-type SWM from 51 to 72, and M5-type SWM from 61 to 87. The pronounced improvement in G2 relative to G1 emphasizes the superior role of reinforcement in resisting crack propagation and enhancing post-crack behavior. This behavior can be attributed to the mesh’s ability to distribute stresses more uniformly and delay structural failure, which becomes increasingly effective as the mesh diameter and surface area coverage increase^50^. Notably, while SWM alone contributes to a modest enhancement in G1, its influence on G2 is considerably more pronounced, indicating its primary contribution lies in mitigating post-crack damage rather than crack initiation. When steel fibers are incorporated alongside SWM, the synergistic effect becomes evident. The G1 values for the SWM and steel fiber combination range from 90 to 99, with the lowest at M40-type SWM and the highest at M5-type SWM (Fig. 16b). Although the synergistic effect shows minimal improvement in G1, consistent with earlier discussions on crack initiation, the trend in G2 is markedly different. For G2 the values demonstrate a significant enhancement, with M40-type SWM ranging from 354 to 394, M30-type SWM from 363 to 413, M20-type SWM from 386 to 448, M10-type SWM from 419 to 469, and M5-type SWM from 435 to 484. The combination of SWM and steel fibers addresses the limitations of each component when used in isolation. SWM predominantly enhances stress distribution and delays macro-crack propagation, whereas steel fibers reinforce the matrix at a micro-scale, improving crack resistance and load redistribution^51^. The complementary reinforcement mechanisms provided by SWM and steel fibers significantly outperform the individual contributions of either component, transforming the brittle failure mode of PAC into a more ductile and resilient response under impact loading^10^. As illustrated in Fig. 16b, an increase in the diameter of SWM from 75 mm to 150 mm led to a corresponding increase in G2 across all types of SWM. The observed behavior is attributed to the increased diameter of the SWM. The larger surface area of SWM enhances stress distribution, resulting in a more uniform dissipation of impact forces^21^. This uniform distribution effectively reduces stress concentrations, thereby delaying the initiation of cracks and subsequent structural failure. SWM with larger diameters provides a more extensive reinforcing framework that effectively impedes crack propagation. The mesh functions by bridging cracks efficiently, resulting in a significant improvement in post-crack performance^21^.

### Impact ductility index

**Fig. 17:**
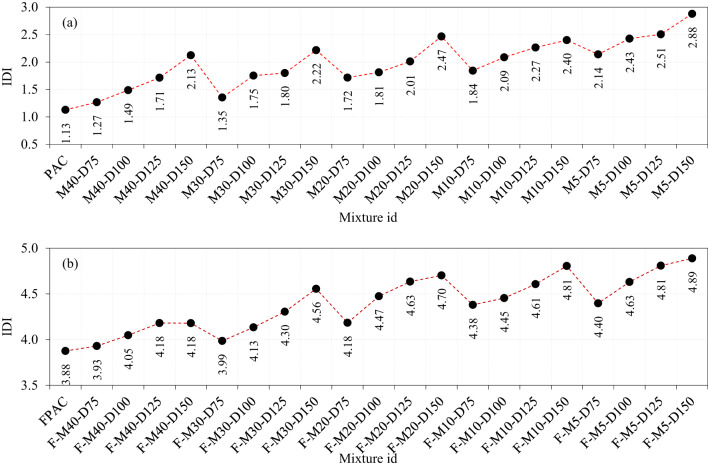
Impact ductility index of the specimens (**a**) SWM, (**b**) SWM + fibers.

The IDI is described as the ratio of G2/G1 which is a measure of a PAC’s ability to undergo plastic deformation under impact loading before failure, with particular emphasis on fibrous PAC. It quantifies the enhancement in energy absorption, toughness, and post-peak behavior attributed to the incorporation of SWM and fibers^10^. The PAC specimen exhibits the lowest IDI of 1.13, primarily due to the absence of fibers, which limits crack bridging and results in accelerated crack propagation, thereby reducing the material’s energy absorption capacity^10^. As shown in Fig. 17a, the IDI values for the specimens with SWM reinforcement vary across different mesh types: from 1.27 to 2.13 for M40-type SWM, 1.35 to 2.22 for M30-type SWM, 1.72 to 2.47 for M20-type SWM, 1.84 to 2.40 for M10-type SWM, and 2.14 to 2.88 for M5-type SWM. A critical observation from these data is that increasing the diameter of the SWM, regardless of the mesh size, consistently leads to a rise in the IDI values. This trend suggests that larger diameter meshes, possibly due to their enhanced capacity for crack bridging and energy dissipation, contribute significantly to the PAC’s overall impact resistance^43^. In comparison, smaller diameter SWM result in less efficient crack management, highlighting the role of SWM diameter in improving the impact ductility of the PAC^10^.

The synergistic effect of SWM and 5D steel fibers on the IDI demonstrated remarkable enhancement across different mixes, with IDI values ranging from 3.93 to 4.18 for M40-type SWM, 3.99 to 4.56 for M30-type SWM, 4.18 to 4.70 for M20-type SWM, 4.38 to 4.81 for M10-type SWM, and 4.40 to 4.89 for M5-type SWM (Fig. 17b). Critically, these findings indicate a clear improvement in impact resistance with increasing diameter of SWM with fibers, with the M5-type SWM exhibiting the highest IDI values (4.40–4.89). This is suggestive of the greater synergy between the steel fibers and M5-type, where the combined effect likely results in better crack bridging, energy dissipation, and delay in failure propagation. The relatively higher IDI values for M5-type SWM can be attributed to the more efficient distribution of fibers and the enhanced interaction between the wire mesh and the geopolymer matrix, providing greater crack resistance.In contrast, the M40-type SWM shows a slightly lower range of IDI values (3.93–4.18), which could reflect the greater stiffness and less flexible nature of the higher-grade mesh, potentially leading to a less favorable interaction with the fibers. While M40-type SWM still enhances impact resistance compared to PAC, the interaction between the SWM and fibers might not be as optimized as in the case of the lower-grade meshes. This could be due to the differences in the mechanical properties of the mesh and its ability to accommodate the deformational capacity of the fibers under impact loading^43^. The previous studies reported the IDI values for the different type of fibrous concrete: 1.45–1.66 by Abid et al.^53^, 9.07–10.64 by Murali et al.^10^, 2.10–2.60 by Murali et al.^19^, 2.6-6.67 (polypropylene fiber) and 1.35 to 1.36 when polypropylene and basalt fibers by yan et al.^54^. It is important to highlight that the observed improvements in IDI, particularly for specimens with M5-type SWM and 5D steel fibers (IDI range: 4.40–4.89), surpass those reported in similar studies involving hybrid fiber-reinforced or mesh-reinforced geopolymer and cementitious composites. Previous research has often demonstrated IDI values below 4.0 in comparable material systems, indicating that the synergistic interaction between the steel wire mesh and the hooked-end steel fibers in our study contributes more significantly to energy dissipation and delayed crack propagation under impact loading. This comparative evaluation not only reinforces the enhanced ductility achieved through our reinforcement strategy but also underscores the novelty and practical relevance of our findings.

### Failure pattern of fibrous PAC

**Fig. 18:**
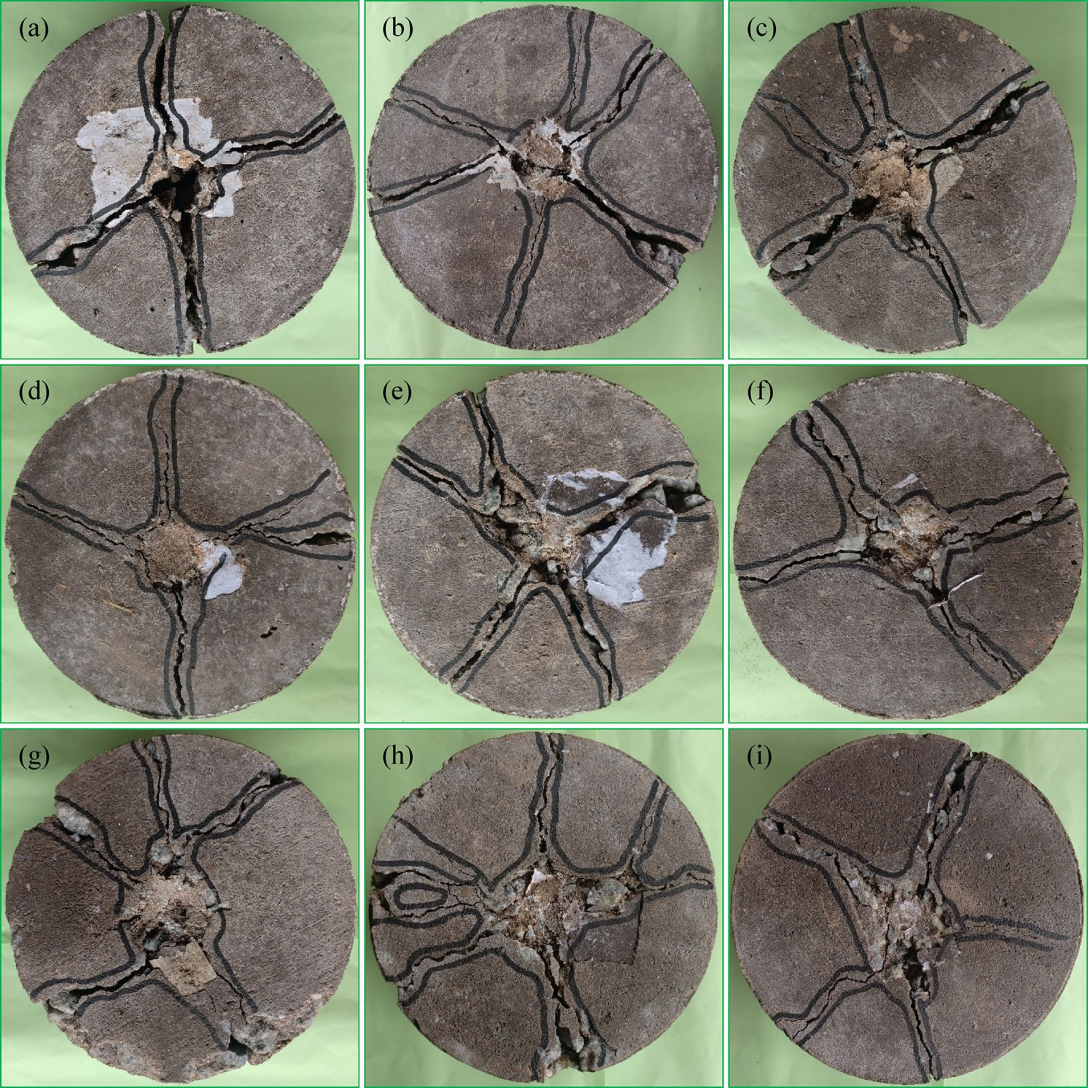
(**a**) PAC, (**b**) M40-D75, (**c**) M40-D100, (**d**) M40-D125, (**e**) M40-D150, (**f**) M5-D75, (**g**) M5-D100, (**h**) M5-D125, (**i**) M5-D150.

The failure patterns observed in fiber-free and fibrous PAC reinforced with SWM of M40 and M5 type and diameters are detailed in Fig. 18. Fiber-free PAC exhibited abrupt and rapid failure following the initiation of cracks, reflecting a brittle fracture mechanism. This behavior required minimal impact strikes to fully propagate cracks through the specimen, resulting in fragmentation into two or three separate pieces (Fig. 18a). In contrast, PAC specimens reinforced with SWM (Fig. 18b-i) displayed a failure mode that, while initially like the brittle fracture of fiber-free PAC, demonstrated significant improvements in impact energy absorption. Notably, specimens incorporating M5-type SWM with mesh diameters increasing from 75 mm to 150 mm exhibited superior performance compared to the M10, M20, M30 and M40 type SWM. This enhancement is linked to the SWM’s capacity to sustain impact loads even after the onset of cracking, indicating a higher deformation capability before ultimate failure. The failure pattern of the specimen remains largely unaffected by variations in the diameter and type of SWM, indicating no significant influence of these parameters. The presence of SWM served to mitigate the rapid propagation of damage, thereby reducing the risk of catastrophic failure. Mechanistically, SWM acted as a barrier to crack propagation^10^, effectively arresting the progression of fractures toward the specimen’s bottom. Larger SWM diameters, particularly 150 mm, provided enhanced resistance^10^, facilitating a transition from brittle to ductile failure modes. Unlike smaller-diameter meshes, these larger configurations prevented the complete disintegration of specimens into multiple fragments^31^. The delayed progression of fractures was primarily attributed to cracks around larger aggregate particles within critical zones, a phenomenon further supported by the SWM’s bridging action. Specimens containing 150-mm diameter SWM exhibited closely spaced cracks on their upper surfaces, highlighting an improved energy dissipation mechanism with the increased number of impact blows. Among the tested configurations, all five SWM with a 150-mm diameter proved most effective at inhibiting crack propagation compared to smaller diameters (75 mm to 125 mm).

**Fig. 19:**
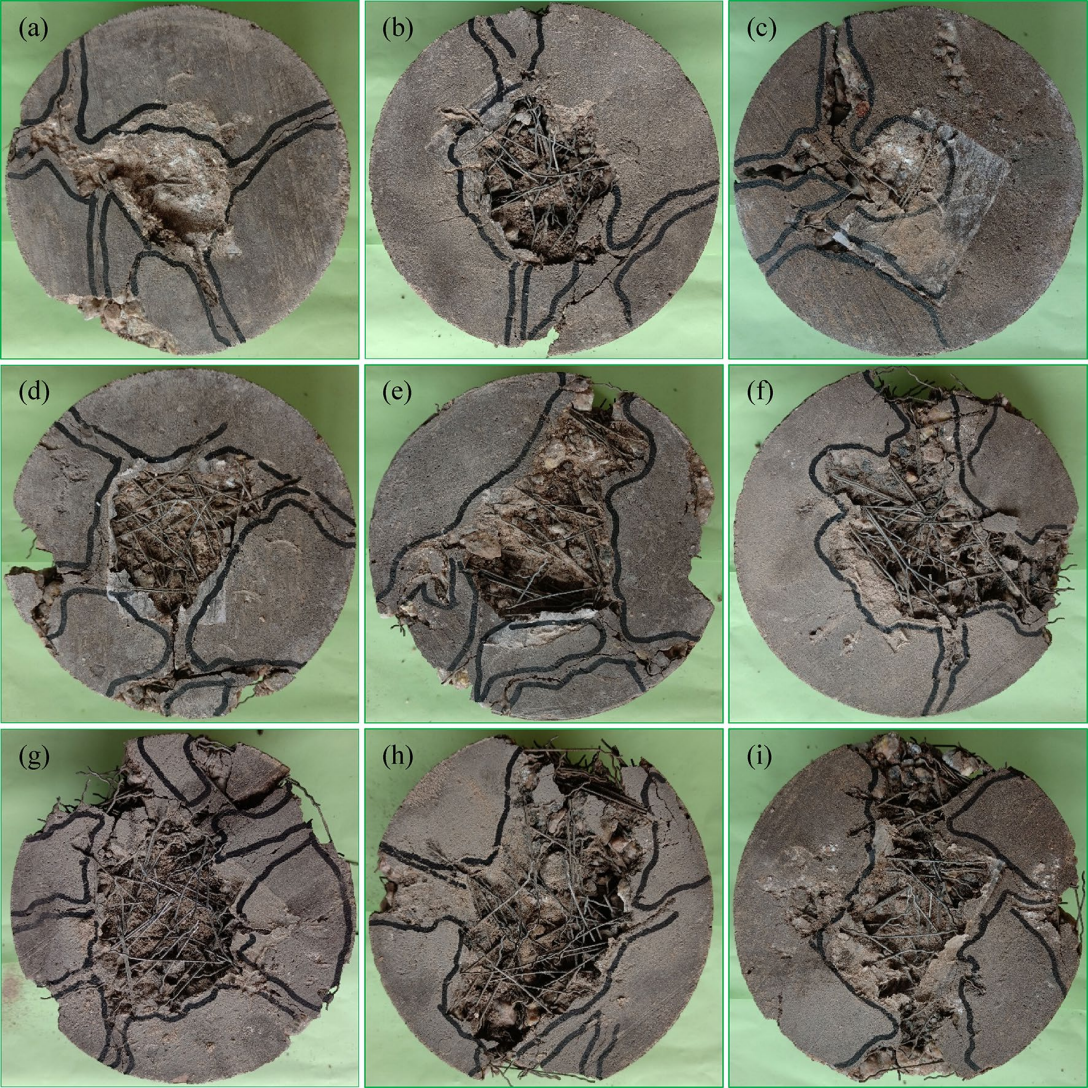
(**a**) FPAC, (**b**) F-M40-D75, (**c**) F-M40-D100, (**d**) F-M40-D125, (**e**) F-M40-D150, (**f**) F-M5-D75, (**g**) F-M5-D100, (**h**) F-M5-D125, (**i**) F-M5-D150.

The synergistic integration of SWM and 5D steel fibers markedly enhanced the impact resistance of fibrous specimens, facilitating a definitive transition towards ductile failure (Fig. 19a-i). Under impact loading, a circular fracture zone formed beneath the steel ball, signifying a progressive increase in impact energy absorption, which was predominantly attributed to the gradual exposure of steel fibers throughout the tested specimens^55^. The growth of this fracture zone was primarily driven by the initiation and propagation of surface cracks of varying magnitudes under repeated impact cycles. With successive impacts, these cracks advanced outward towards the specimen’s periphery and downward to its lower surface. Steel fibers were instrumental in mitigating crack propagation by effectively bridging the crack interfaces and maintaining structural cohesion^56^. However, over time, the adhesion between the fibers, SWM, and the geopolymer matrix diminished, ultimately resulting in failure characterized by fiber pull-out. Among the configurations examined, the inclusion of SWM with a 150 mm diameter in conjunction with steel fibers exhibited the most pronounced efficiency in restricting crack propagation towards the bottom surface, highlighting the advantageous role of larger SWM diameters in reinforcing structural stability. The M5 type SWM demonstrated the highest crack propagation resistance, followed sequentially by M10, M20, M30, and M40 combinations with steel fibers, underscoring the critical importance of selecting the appropriate SWM type and size to optimize the performance of fiber-reinforced geopolymer composites. While the presence of SWM contributed to decelerating crack progression, the role of steel fibers was notably more significant in mitigating crack growth and preserving the structural integrity of the specimens^57^. SWM with diameters exceeding 150 mm effectively served as barriers against downward crack propagation, significantly enhancing resistance and ensuring the stability of specimens under impact loading. In contrast, a 75 mm diameter SWM failed to adequately impede crack advancement, demonstrating limited efficacy as a reinforcing element. These findings underscore the necessity of achieving an optimal balance between SWM diameter and steel fiber incorporation to enhance impact resistance^10^ and crack control in fiber-reinforced geopolymer composites.

### Failure plane of the tested specimens

**Fig. 20:**
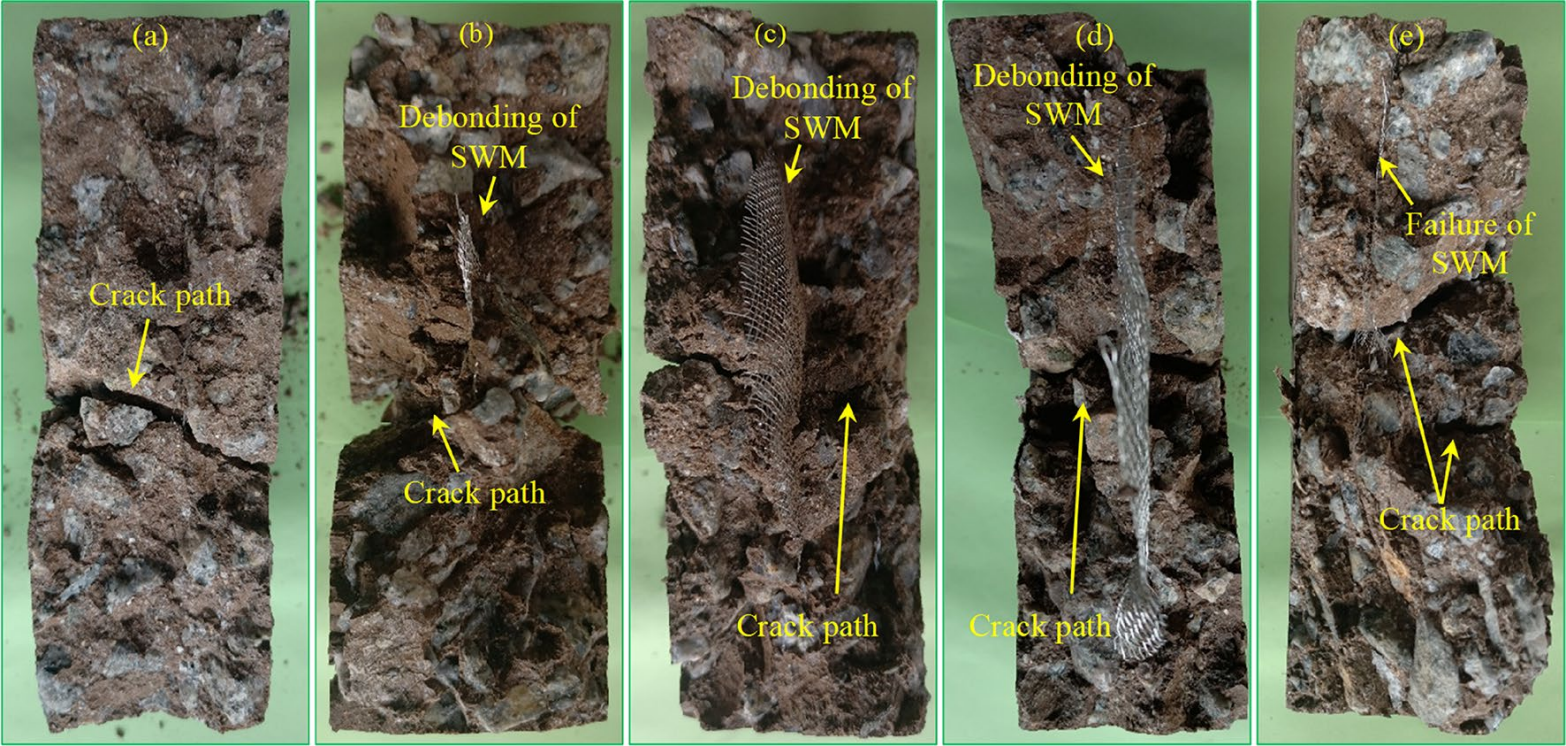
Failure plane of the specimens (**a**) PAC, (**b**) M10-D75, (**c**) M10-D100, (**d**) M10-D125 and (**e**) M10-D150.

**Fig. 21:**
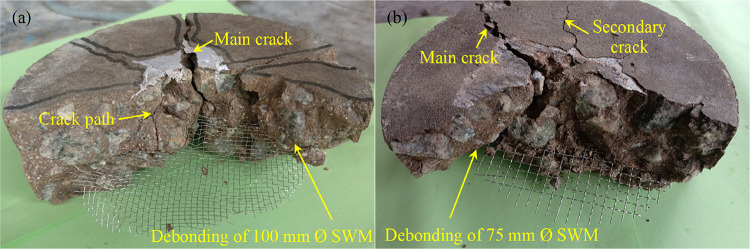
Debonding of SWM (**a**) M10-D100 and (**b**) M5-D75.

Figure 20 depicts the failure planes of nonfibrous PAC specimens incorporating various diameters of M10 type insertion. Figure 20a illustrates that the crack path in PAC specimens without SWM consists of a single prominent macro-crack, indicating that PAC with geopolymer grout, in the absence of reinforcement, predominantly exhibits localized failure within the impact zone. The cracks are generally linear and propagate at an accelerated rate. The matrix composition, consisting of red clay, slag, and fly ash, exhibits a significant influence on crack propagation mechanisms, with each component imparting distinct mechanical behaviors. Red clay, characterized by its high brittleness, accelerates crack propagation, making the matrix more prone to localized failure^58^. The addition of SWM plays a pivotal role in altering crack dynamics by introducing effective crack-arresting mechanisms. Unlike unreinforced matrices that typically develop a single dominant crack, SWM reinforces the matrix, resulting in a more distributed failure plane (Fig. 20b-e). This behavior highlights the efficacy of SWM in promoting energy dissipation through crack branching and redistribution. Furthermore, the impact of SWM diameter on energy dissipation is noteworthy. Specimens reinforced with a 75 mm diameter SWM display limited energy dissipation, suggesting insufficient reinforcement coverage or crack-bridging capacity (Fig. 20b). Conversely, a 150 mm diameter SWM demonstrates superior performance, with significantly higher energy dissipation attributed to enhanced reinforcement-matrix interaction and a broader dissipation area (Fig. 20e). These observations underscore the critical balance required in selecting SWM dimensions to optimize crack deflection, energy absorption, and overall structural resilience. As illustrated in Fig. 21, debonding was observed in the M10 and M5 types of SWM rather than tearing the mesh by the propagating cracks. This behavior can be ascribed to the smooth surface texture of the steel wires, which offers limited mechanical interlocking with the surrounding matrix, thereby reducing the bond strength. Moreover, the considerable stiffness contrast between the SWM and the geopolymer matrix exacerbates this effect. Under applied stress, the mismatch in strain responses generates interfacial shear stresses, which ultimately result in debonding at the matrix-reinforcement interface^59^.

The incorporation of steel fibers and SWM markedly improves the energy absorption capacity of the composite, showcasing a highly effective interaction between these reinforcement elements^46^. In comparison to specimens without fibers, which exhibit immediate structural collapse upon impact, the reinforced composites demonstrate superior post-impact integrity^56^. This enhanced performance is attributed to the synergistic bridging effects of both SWM and steel fibers, which serve to suppress crack propagation and promote efficient energy dissipation. Reinforced specimens exhibit failure planes with multiple secondary cracks^60,61^, signifying distributed energy absorption, whereas fiber-free counterparts tend to fail through singular, clean fractures, and rapid disintegration under equivalent conditions. Additionally, the failure mechanisms observed in fibrous specimens are characterized by fiber pull-out and SWM deformation, which play a critical role in dissipating and redistributing impact energy^10^. These features stand in stark contrast to the brittle fracture behavior typical of unreinforced composites, where the absence of such mechanisms results in abrupt failure^57^. Importantly, all fibrous specimens maintained structural cohesion, avoiding complete separation into two fragments even under substantial impact forces. This outcome highlights the combined contribution of steel fibers and SWM in improving the ductility and impact resistance of the composite, underscoring their superiority over unreinforced systems^10,62^.

While the introduction of SWM and steel fibers in PAC significantly enhances energy absorption, impact resistance, and post-cracking behavior, these benefits may be accompanied by certain trade-offs. Notably, the incorporation of larger-diameter SWM (e.g., 150 mm) and high-performance 5D steel fibers could increase overall material costs and raise concerns regarding the availability and fabrication complexity of customized mesh types, especially M5- and M10-type configurations. Additionally, the handling and placement of dense reinforcement may affect workability and construction efficiency. Therefore, practical implementation should consider these economic and logistical constraints to ensure that performance gains align with project-specific cost and resource considerations.

## Microstructure analysis

### SEM analysis

**Fig. 22:**
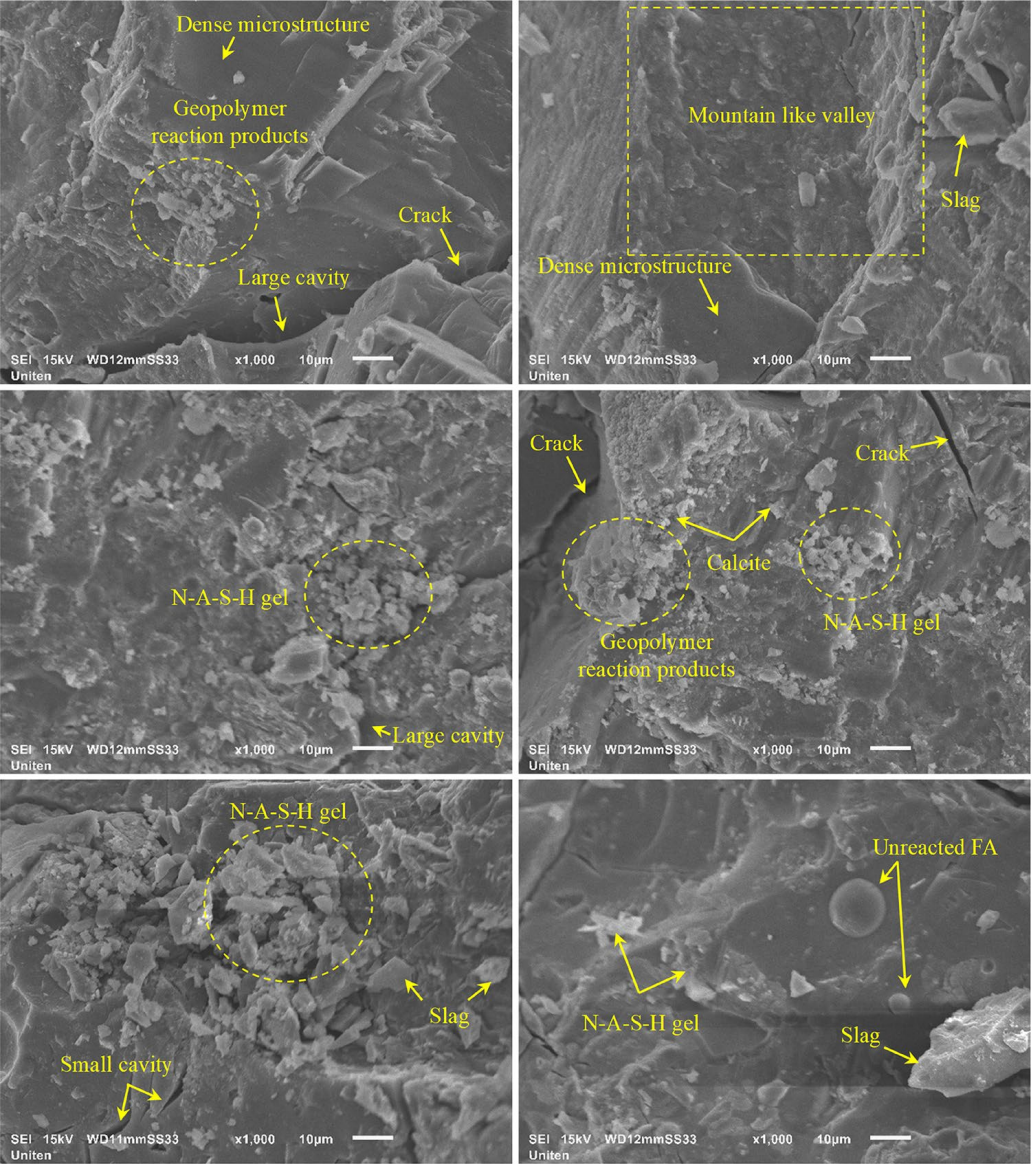
SEM analysis of the geopolymer grout-based PAC sample.

An SEM analysis was conducted on geopolymer concrete containing RC, fly ash and slag, as depicted in Fig. 22. The microstructural examination reveals a sparse presence of spherical particles, indicating the existence of unreacted fly ash and slag. This observation suggests incomplete geopolymerization, likely resulting from insufficient polymerization reactions^63^. The microstructural analysis of geopolymer concrete indicates enhanced packing efficiency, increasing amorphous geopolymer gel formation and compressive strength. This improvement results from the efficient depolymerization of fly ash aluminosilicates, releasing aluminate and silicate species. Alkaline activation facilitates monomer condensation and polymerization, forming a robust inorganic polymer matrix^64^. Mahmoud et al.^65^ reported that the presence of geopolymeric gels is correlated with improved bond strength, enhanced mechanical properties, and greater homogeneity in geopolymer mixtures. Additionally, the higher incorporation of slag greatly enhanced the CaO content within the mixture, promoting the formation of a higher quantity of N–A–S–H gels observed in the samples^66^. Sidhu and Kumar^67^ stated that in fly ash-based geopolymers incorporating slag, geopolymeric phases such as C-A-S-H and N-A-S-H gels coexist with C-S-H gel, which is characterized by a low Ca/Si ratio. The SEM analysis reveals a negligible existence of unreacted fly ash particles. The higher slag content substantially improves the activation of fly ash, thereby reducing the occurrence of unreacted particles and minimizing the formation of irregular pores^68,69^. The formation of cracks in the specimens appears to be attributable to the presence of residual unreacted fly ash combined with the evaporation of free water during the ageing and curing processes, thereby facilitating the development of microcracks. The progressive propagation of microcracks leads to localized microstructural degradation, consequently reducing the mechanical strength^67^.

### XRD analysis of PAC with geopolymer grout

**Fig. 23:**
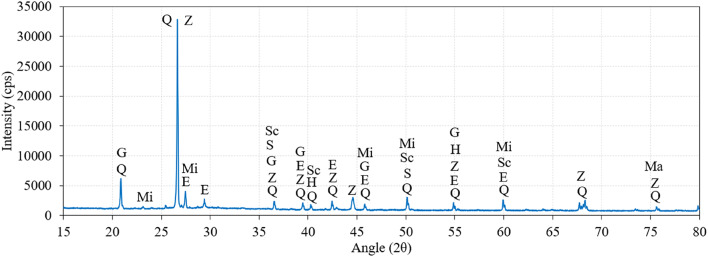
XRD analysis of the PAC samples (G: Gypsum, Mi: Microcline, Z: Zeolite, E: Ettringite, S: Suolunite, Sc: Scolecite, Ma: Magnetite).

The XRD analysis of the PAC with geopolymer grout is depicted in Fig. 23. The peaks of Gypsum, Microcline, Zeolite, Ettringite, Suolunite, Scolecite and Magnetite were observed. The investigation of geopolymer concrete synthesized from red clay, fly ash, and slag employed XRD to identify distinct mineral constituents. Notably, gypsum and quartz were discerned at a 2θ angle of 20.86°, signifying their functions as a setting-retarding agent and a strength-enhancing component, respectively. Gypsum is recognized for its capacity to improve workability and initial performance metrics, whereas quartz contributes significantly to the mechanical integrity of the composite. Furthermore, quartz was observed in association with zeolite at 26.64° 2θ. The presence of zeolite is particularly significant due to its pozzolanic properties, which facilitate long-term durability and strength enhancements through chemical reactions that produce supplementary binding phases. Cai et al.^70^ identified a characteristic “hump” in the X-ray diffraction pattern at 27° to 29°, indicating the presence of geopolymers. This hump signifies an amorphous silica-aluminate gel phase, suggesting the possible formation of calcium-aluminosilicate hydrates (C-A-S-H) and sodium-aluminosilicate hydrates (N-A-S-H). The primary crystalline phases observed include mullite, quartz, and calcite, with mullite and quartz originating from fly ash. Criado et al.^71^ reported the persistence of trace amounts of peaks corresponding to mullite and quartz, indicating that the raw materials had not undergone complete reaction and consumption during the process.

## Conclusions

This study examined the enhancement of impact strength in sustainable PAC utilizing a RC, slag, and fly ash-based geopolymer grout, with a focus on the synergistic contributions of five different steel wire meshes of varying diameters and 5D steel fibers. The subsequent conclusions are drawn from the comprehensive investigation.

1. The M40-type SWM (75–150 mm) in PAC with geopolymer grout led to marginal G1 improvements (1.07–1.14 times the control) but significantly enhanced G2 values (1.20–2.15 times), highlighting its role in post-cracking behavior and energy dissipation. Combining SWM and steel fibers consistently improved G1 (1.04–1.09 times) and G2 (1.06–1.18 times) by enhancing energy absorption and impact resistance.
2. M30-type SWM led to modest G1 gains (1.15–1.25 times) with minimal sensitivity to diameter, while G2 improved significantly (1.40–2.47 times), peaking at 150 mm. Adding steel fibers with SWM consistently enhanced G1 (1.04–1.06 times) and G2 (1.08–1.24 times), highlighting the importance of SWM diameter in optimizing energy absorption and structural resilience.
3. In PAC with M20-type SWM, larger diameters (75–150 mm) improved G1 (1.16–1.26 times) and significantly enhanced G2 (1.75–2.75 times) through better energy absorption and crack resistance. Adding fibers further boosted G1 (1.07–1.11 times) and G2 (1.16–1.34 times), with greater benefits at larger diameters.
4. Specimens with SWM alone showed limited G1 values (25–31), while G2 values improved notably across SWM types, increasing from 32 (M40) to 87 (M5). Adding steel fibers significantly boosted G1 (90–99) and greatly enhanced G2, ranging from 354 (M40) to 484 (M5), with M5-type SWM delivering the highest performance.
5. The synergy between SWM and 5D steel fibers significantly improves IDI, with the highest values (4.40–4.89) in M5-type SWM specimens. In contrast, M40-type SWM yields lower IDI (3.93–4.18), highlighting the role of SWM flexibility and fiber interaction in enhancing impact resistance and delaying failure under impact loading.
6. Adding SWM to PAC enhances impact resistance by limiting crack propagation, with larger diameters (especially 150 mm) boosting energy absorption and promoting ductile failure. SWM’s bridging action prevents sudden failure, while combining it with 5D steel fibers further improves cohesion and delays crack growth.
7. SEM analysis of geopolymer concrete with fly ash and slag shows unreacted particles, indicating incomplete geopolymerization. Improved packing efficiency enhances amorphous geopolymer gel formation, leading to increased compressive strength due to effective depolymerization of fly ash aluminosilicates.
8. XRD analysis of PAC-based geopolymer grout identified peaks for Gypsum, Microcline, Zeolite, Ettringite, Sulfonite, Scolecite, and Magnetite. Gypsum (20.86° 2θ) serves as a setting-retarding agent, while quartz enhances strength. Zeolite (26.64° 2θ) exhibits pozzolanic properties, promoting additional binding phases and improving long-term durability and strength.

### Recommendation for the future works

- The combined use of fly ash, slag, and red clay as SCMs may reduce pore solution alkalinity. This reduction in alkalinity could weaken steel passivation, increasing corrosion risk under moisture, chloride ingress, or carbonation. Further investigations are needed to explore these effects as part of future research.
- While the synergistic effect of SWM and 5D steel fibers is evident, the precise interaction mechanisms at the microstructural level (e.g., fiber orientation, bond strength, and bridging action) are not fully explored.
- The study is limited to laboratory-scale specimens; further research is needed to assess the scalability of the PAC system, including its constructability, placement methods, and compatibility with current construction practices.
- While the study investigates five SWM sizes (M40 to M5) with a fixed fiber dosage (2.5% by volume), it does not fully explore the optimal mesh configuration, spacing, or fiber content for various structural applications. Future work should conduct parametric studies to determine ideal combinations for enhanced mechanical and energy dissipation performance.
- The mechanical advantages of SWM and fiber-reinforced geopolymer PAC offer a strong technical foundation for inclusion in construction standards. Regulatory bodies can explore pilot projects and certifications, especially for high-impact applications like barriers, pavements, and blast-resistant structures, supporting its adoption in resilience policies and green building systems.
- Given the enhanced impact resistance, crack control, and energy absorption achieved, this study supports large-scale construction trials in high-risk zones, including: transportation infrastructure (e.g., railway sleepers, bridge decks, highway barriers), defense structures (e.g., bunkers, blast walls), industrial heavy-duty flooring, and precast elements for modular construction systems.

## Data Availability

Data is provided and available within the manuscript.
